# Maternal-prenatal gut microbiome-systemic metabolome perturbations and T_H_2-skewed immunity link to offspring gut microbiome disruption and atopic dermatitis susceptibility

**DOI:** 10.1186/s13073-026-01655-5

**Published:** 2026-04-17

**Authors:** Daniel Zhi Wei Ng, Gaik Chin Yap, Carina Jing Xuan Tay, Chiung-Hui Huang, Siyan Zhao, Adrian Low, Elizabeth Huiwen Tham, Evelyn Xiu Ling Loo, Lynette P. Shek, Anne Goh, Kok Wee Chong, Si Hui Goh, Zai Ru Cheng, Hugo P. S. Van Bever, Oon Hoe Teoh, Yung Seng Lee, Fabian Yap, Kok Hian Tan, Yap-Seng Chong, Shiao-Yng Chan, Johan Gunnar Eriksson, Keith M. Godfrey, Christophe Lay, Jan Knol, Stephan C. Schuster, Jun Shi Lai, Mary Foong-Fong Chong, Jonathan Wei Jie Lee, Bee Wah Lee, Eric Chun Yong Chan, Le Duc Huy Ta

**Affiliations:** 1 Department of Pharmacy and Pharmaceutical Sciences, National University of Singapore, 18 Science Drive 4, Singapore, 117543 Singapore; 2 Department of Paediatrics, National University of Singapore, Yong Loo Lin School of Medicine, 1E Kent Ridge Road, NUHS Tower Block, Level 12, Singapore, 119228 Singapore; 3 Department of Medicine, Yong Loo Lin School of Medicine, National University of Singapore, MD6 Centre for Translational Medicine, 14 Medical Drive, Singapore, 117599 Singapore; 4 Division of Allergy & Immunology, Khoo Teck Puat-National University Children’s Medical Institute, National University Health System, Singapore, Singapore; 5 Institute for Human Development and Potential (IHDP), Agency for Science, Technology and Research (A*STAR), Singapore, Singapore; 6 Human Potential Translational Research Programme, Yong Loo Lin School of Medicine, NUS, Singapore, Singapore; 7 Allergy Service, Department of Paediatrics, KK Women’s and Children’s Hospital, Singapore, Singapore; 8 Respiratory Medicine Service, Department of Paediatrics, KK Women’s and Children’s Hospital, Singapore, Singapore; 9 Division of Paediatric Endocrinology, Khoo Teck Puat-National University Children’s Medical Institute, National University Health System, Singapore, Singapore; 10 Department of Paediatrics Endocrinology, KK Women’s and Children’s Hospital, Singapore, Singapore; 11 Department of Maternal Fetal Medicine, KK Women’s and Children’s Hospital, Singapore, Singapore; 12 Department of Obstetrics & Gynaecology, National University Health System, Yong Loo Lin School of Medicine, National University of Singapore, Singapore, Singapore; 13 Folkhälsan Research Center, Helsinki, Finland; 14 Department of General Practice and Primary Health Care, University of Helsinki, Helsinki, Finland; 15 MRC Lifecourse Epidemiology Centre and NIHR Southampton Biomedical Research Centre, University of Southampton and University Hospital Southampton NHS Foundation Trust, Southampton, UK; 16 Danone Research and Innovation, Precision Nutrition D-lab, Singapore, Singapore; 17 Danone Research and Innovation, Utrecht Center, Utrecht, The Netherlands; 18 APC Microbiome Ireland, University College Cork, Cork, Ireland; 19 Singapore Centre for Environmental Life Sciences Engineering, Nanyang Technological University, Singapore, Singapore; 20 Saw Swee Hock School of Public Health, National University of Singapore, 12 Science Drive 2, #09-01Q, Singapore, 117549 Singapore; 21 Institute for Health Innovation and Technology (iHealthtech), National University of Singapore, E7, 15 Kent Ridge Crescent, Singapore, 119276 Singapore; 22 Division of Gastroenterology & Hepatology, Department of Medicine, National University Hospital, Singapore, Singapore

**Keywords:** Paediatric allergy, Prenatal-maternal biological signature, Multi-omic analyses, Developmental biology, Mother–offspring interface, S-PRESTO Birth Cohort, Atopic dermatitis, Microbiome-metabolome-immunity

## Abstract

**Background:**

Emerging evidence suggests that maternal-prenatal gut microbiome disturbances shape offspring allergic outcomes through modulation of the in utero immune environment. Yet, no comprehensive clinical studies in human mother–offspring dyads have deconvoluted the maternal-prenatal gut microbiome and systemic immune-metabolome signatures underlying offspring allergic predisposition.

**Methods:**

We performed a longitudinal nested case–control study involving 128 well-characterized mother–offspring dyads from defined cases (offspring with atopic dermatitis (AD); *n* = 64) and controls (offspring without AD; *n* = 64). Maternal stool and blood samples were collected at multiple time points during gestation for multi-omic profiling. Structural and functional gut microbiome composition was characterized via metagenomic sequencing, while systemic metabolome and serum immune milieu were profiled using targeted plasma metabolomics and Olink proximity extension assays, respectively. In offspring early-life, stool samples were collected longitudinally up to 6 months of age for gut microbiome and metabolome analyses.

**Results:**

Mothers of AD infants exhibited longitudinal enrichments of gut *Klebsiella pneumoniae*, *Roseburia intestinalis*, *Clostridioides difficile* and *Bilophila* sp. 4_1_30, alongside depletions in gut *Clostridium* sp. CAG:678*, Romboutsia timonensis*, *Akkermansia muciniphila*, *Blautia hansenii* and *Alistipes ihumii* during pregnancy. These taxonomic shifts were associated with systemic metabolomic alterations, including elevated branched-chain amino acids and immune-related metabolites (e.g., creatine, ornithine), and a concurrent pro-inflammatory T_H_2-skewed immunological milieu marked by increased interleukin-4 (IL-4) and IL-5 and decreased CXCL11. In early life, AD infants harbored a dysbiotic gut microbiome characterized by persistent enrichments of potentially pathogenic *Escherichia coli* and *K. pneumoniae*, along with depletion of short chain fatty acid-producing *Bacteroides* species and beneficial colonizers*.* Integrated multi-omic analyses across the prenatal-postnatal axis indicated that the impaired establishment of gut microbiome in AD infants may, in part, be attributed to the (1) potential transmission of maternally originated *Klebsiella* and (2) immunomodulatory effects of a maternal-prenatal pro-inflammatory, T_H_2-skewed milieu during gestation.

**Conclusions:**

Our study uncovers a distinct maternal-prenatal gut microbiome and systemic metabolome–immune signature that predisposes offspring to AD by disrupting early-life gut microbial establishment. These findings highlight the gestational period as a critical window for preventive strategies targeting the maternal microbiome or systemic immune-metabolic axes to mitigate allergic disease susceptibility in offspring.

**Trial registration:**

This study is registered at ClinicalTrials.gov (NCT 03531658).

**Supplementary Information:**

The online version contains supplementary material available at 10.1186/s13073-026-01655-5.

## Background

Early onset childhood atopic dermatitis (AD) incurs a significant disease burden, affecting 20% of children globally [1]. With symptoms typically emerging before 2 years of age [2], the condition places substantial economic and emotional burden on caregivers [3]. The pathophysiology underlying infant AD development is multifaceted, involving the complex interplay of epidermal dysfunction [4], genetic predisposition [5], environmental factors, and immune system dysregulation [4].

Recent studies have alluded to the potential influence exerted by the maternal gut microbiome during pregnancy on offspring allergic outcomes through modulation of the in utero immune environment [6]. During pregnancy, the maternal gut microbiome undergoes profound remodeling [7], priming the maternal-foetal immunological environment through systemic exposure to a dynamic and fluctuating range of gut microbiome-derived metabolites [8]. Presently, there is a lack of comprehensive longitudinal multi-omic clinical studies in human mother–offspring dyads which investigate the dynamic interaction between the prenatal gut microbiome, systemic metabolome and immune interactions preceding offspring allergic disease development. Understanding the complex interplay between the biological signatures is crucial for the formulation of clinical recommendations during pregnancy to reduce offspring allergy risk.

**Fig. 1:**
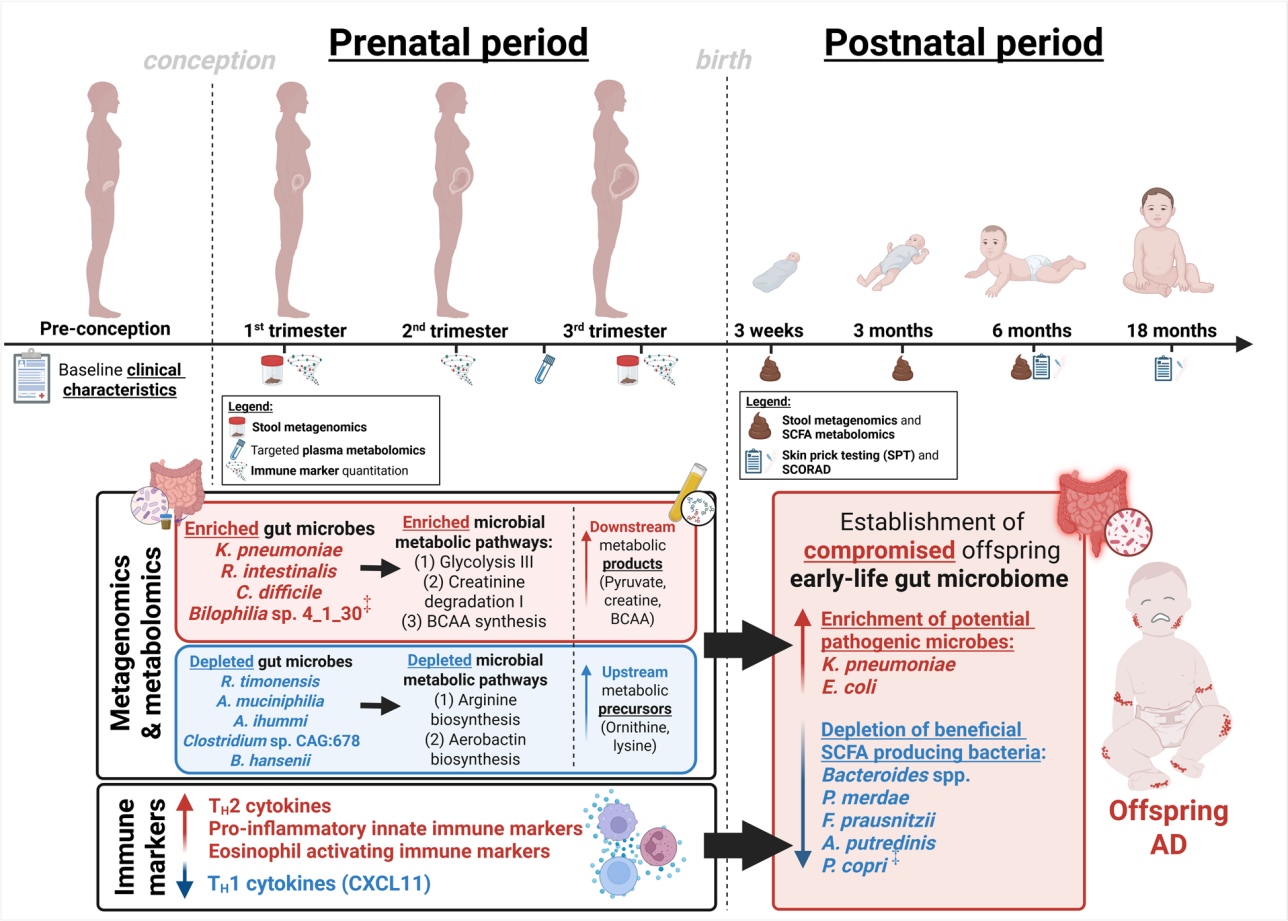
Maternal-prenatal biological signature that predisposes offspring to AD. We identified significant perturbations in maternal gut microbiota and their associated functional gene carriage, alongside a corresponding systemic immune-metabolome signature in mothers of AD offspring. This maternal signature was associated with a compromised early-life gut microbiome in AD offspring, characterized by enrichments in potentially pathogenic *Enterobacteriaceae* and depletion of short chain fatty acid (SCFA) producing bacteria species. All reported associations remained significant after FDR correction across both maternal and offspring features. The symbol ‡ denotes taxa exhibiting nominal longitudinal significance that did not meet the FDR threshold

In this study, we performed an integrated and longitudinal multi-omic nested case–control study derived from the clinically well-characterized S-PRESTO (Singapore PREconception Study of Long-Term Maternal and Child Outcomes) mother–offspring cohort to comprehensively interrogate prenatal and early-life influences that underscore offspring AD development. Our study aims to provide critical evidence that the maternal-prenatal biological signature serves as a key inciting factor that triggers a cascade of events that shape offspring gut microbiome-metabolome trajectories and contribute to subsequent predisposition to AD (Fig. 1).

## Methods

### Sample cohort

Mother and offspring pairs in both case (offspring AD) and control groups (*n* = 64 in each group) were drawn from the original S-PRESTO cohort [9] (https://gustodatavault.sg/about/spresto). The S-PRESTO cohort is a multiethnic preconception cohort that recruited Chinese, Malay or Indian women aged 18–35 years between February 2015 and April 2018 [9]. The study design, participant eligibility, inclusion and exclusion criteria of the recruited participants have been comprehensively described [9]. Ethical approval was obtained from the SingHealth Centralised Institutional Review Board (reference 2014/692/D). This study is registered at ClinicalTrials.gov (NCT 03531658).

Of the 1,039 women initially recruited, 373 women remained in the study and gave birth to singletons [9]. A total of 353 mother–child dyads were enrolled and monitored longitudinally up to the child’s second year of age, with 20 participants lost to follow-up over the course of the study. Maternal sociodemographic characteristics and metabolic health parameters were collected during the initial preconception visit. Throughout pregnancy and the postnatal period, detailed clinical histories were obtained for both mothers and their infants. Scheduled prenatal study visits took place during gestational weeks 6–8, 11–13, 18–21, 24–26, 27–28, and 34–36 to facilitate comprehensive clinical evaluation and biological sample collection. Following birth, children were assessed at 1, 3, and 6 weeks, and at 3, 6, 9, 12, 18, 24, and 36 months to track developmental and health outcomes.

### Selection of mother and infant pairs in AD and control group

In this nested case–control study, we selected mother–offspring dyads in the AD group (*n* = 64) based on diagnosis of AD in offspring. Skin examination was performed by research coordinators at months 3 and 12 and by doctors at months 6 and 18 using the Hanifin & Rajka criteria [10] for evaluation of AD presentation in offspring. Interviewer-administered questionnaires captured physician’s diagnosis of offspring AD as determined by a positive answer to the question: “Has your child ever been diagnosed with eczema? Eczema—a medical condition where the skin is red, dry, scaly, itchy and sore”. Assessment of AD severity was determined by SCORing Atopic Dermatitis (SCORAD) grading scale. The 64 mother–offspring pairs in the AD group were selected based on AD infants having the highest SCORAD scores between ages 6 and 18 months.

To minimize bias between groups, mother–offspring dyads in the control group were matched to those in the AD group based on ethnicity, maternal pre-conception body mass index (BMI), antibiotic use during pregnancy and during labour, and offspring mode of delivery.

### Collection of maternal dietary data

Maternal dietary intakes were recorded by trained research staff using a validated, 92-item, semiquantitative food frequency questionnaire (FFQ) [11] during the mothers’ first pre-conception visit and at third (34–36 weeks) trimester of pregnancy. For each FFQ item, frequency of consumption over the past month was indicated following these options: ‘never/rarely’, ‘frequency per month’, ‘frequency per week’ or ‘frequency per day’. Participants were provided with picture aids of various food portion sizes and standard-sized household utensils to gauge the average amount consumed. Daily caloric and nutrient intakes were subsequently estimated using a local food composition database.

### Collection of stool and plasma samples

Biosamples (plasma and serum samples) were collected from mothers during clinical visits and stored in aliquots at −80 °C until further analyses. Plasma samples were drawn from mothers in the third (27–28 weeks) trimester of pregnancy for targeted metabolomics analysis (Bevital, Bergen, Norway, http://www.bevital.no). Serum samples were also collected at five pregnancy timepoints (6–8, 11–13, 18–21, 27–28 and 34–36 weeks) for targeted quantitation of immune markers by Olink Target 96 inflammation panel (Olink Proteomics, Uppsala, Sweden).

For stool sample collection, each mother was provided with a sterile stool collection kit that was couriered to the laboratory once the sample was collected. Fresh faecal samples were collected (1) from the mother during pregnancy and (2) the offspring by their parents using the stool collection kits before immediate storage at − 20 °C freezer. Samples were then transported to the laboratory in cold chain within 20 h of sample collection for aliquoting and subsequently stored at − 80 °C until further analysis (metagenomics and/or stool metabolomics). Stool samples were collected from mothers in the first (11–13 weeks) and third (34–36 weeks) trimesters of pregnancy and from offspring at 3 weeks, 3 months and 6 months of age. Notably, stool samples collected from participants in each group during postnatal timepoints were smaller than the overall number of cases and controls (*n* = 64 in each group). Stool samples were subjected to in-house metagenomics analyses (mother and offspring) and commercial targeted metabolomics analyses (offspring only) by Metabolon (Durham, North Carolina, https://www.metabolon.com/).

### Nucleic acid extraction, metagenomics sequencing and bioinformatics

Nucleic acid extraction, metagenomic sequencing, and downstream bioinformatic processing were conducted following protocols previously described [12], with modifications where applicable. Briefly, approximately 100–150 mg of stool material were used to extract genomic DNA with the ZR fecal DNA MicroPrep kit (Zymo Research, USA) following the manufacturer’s protocol. For each sample, a sequencing library was prepared with Illumina’s Truseq nano DNA library preparation kit (Illumina, San Diego, USA). DNA was fragmented to approximately 450 bp using Covaris E220 and was uniquely tagged with one of Illumina’s TruSeq HT DNA barcodes to allow for multiplexed sequencing. The finished libraries were quantitated with Invitrogen’s Picogreen assay, and the average library size was assessed using Bioanalyzer 2100, DNA 7500 chip (Agilent). Library concentrations were adjusted to 4 nM and validated by qPCR on a ViiA-7 real-time thermocycler (Applied Biosystems), using primers specified in Illumina’s qPCR protocol and the PhiX control library as a standard. Finally, libraries were pooled in equimolar amounts and sequenced on an Illumina HiSeq2500 in rapid mode, generating 250 bp paired-end reads.

The Illumina metagenomic reads were first trimmed for adapters and quality using cutadapt-1.8.1, with the parameters “-q 20 –trim-n –minimum-length 30 –match-read-wildcards” [13]. The resulting reads were then aligned to the hg19 human reference genome using bowtie2.1.0, applying the “–very-sensitive-local” setting for optimal sensitivity [14]. Reads that could not be confidently matched to the human genome—identified using the “–un-conc” option—were separated and treated as non-host reads. These non-host reads were subsequently aligned to the NCBI non-redundant protein database using Diamond version 0.8.5 [15]. Microbial taxonomic classification was performed using the Lowest-Common-Ancestor (LCA) algorithm in MEGAN6 version 6.4.19, with parameters set to maxmatches = 25 and minscore = 100, and a minsupport of 100 [16]. Species-stratified functional pathway analysis was performed to assess longitudinal differences in MetaCyc pathway abundances at the species level. Bacterial taxa that were longitudinally differentially abundant between mothers in the AD and control groups were first identified, and HUMAnN 3 [17] was then used to map taxon-specific gene family abundances to MetaCyc pathways, yielding quantitative pathway abundance estimates for each species–pathway combination per sample. These species-resolved pathway abundance profiles were used to evaluate differences in functional metagenomic potential at the species–pathway level.

### Targeted maternal plasma metabolomics during pregnancy

Amino acids (AA), carboxylic acids, tri-carboxylic acid cycle metabolites and intermediates in maternal plasma at the third trimester of pregnancy were quantitated utilizing a commercial gas chromatography tandem mass-spectrometry (GC–MS/MS) platform developed and validated by Bevital (Bergen, Norway, http://www.bevital.no).

Briefly, plasma samples were subjected to liquid–liquid extraction by mixing with dithioerythritol (containing deuterated internal standard (IS)), ethanol, and isooctane/chloroform before centrifugation and extraction of the aqueous phase. The resultant aqueous fraction was derivatized by mixing with ethanol, water, pyridine, and methylchloroformate in toluene prior to GC–MS/MS analyses. An Agilent 7890B GC system coupled to an Agilent 7010 GC–MS triple quadrupole mass spectrometer was used in electron ionization mode for GC–MS/MS analyses. Helium was used as the carrier gas at a constant rate of 1.2 mL/min and a Varian CP Sil 24-CB low-bleed/MS capillary column, 15 m (length) × 0.25 mm (internal diameter) × 0.25 μm (film thickness), was used as the stationary phase for chromatographic separation of analytes over a pre-set temperature gradient. The interface temperature was 290 °C, the source temperature was 250 °C, and the collision energy was set at 70 eV. Analyte concentrations were calculated by dividing the peak area of analyte by the area of the corresponding IS and comparing the peak area ratio calculated with the area ratios obtained from calibrator plasma which had been spiked with known analyte concentrations. Plasma metabolomics data were reported as concentration of µmol per liter of plasma (µM).

### Targeted metabolomic analyses of stool SCFA in offspring

Stool SCFA in offspring were quantitated by Metabolon, Inc (Durham, North Carolina, https://www.metabolon.com/) using a commercial liquid chromatography-tandem mass spectrometry (LC–MS/MS) platform (Metabolon method TAM135). Stool samples were stored at −80 °C prior to LC–MS/MS analysis.

Briefly, stool samples were spiked with SCFA IS and protein precipitated with methanol before centrifugation. Supernatant was subsequently collected and derivatized prior to injection into a C_18_ reversed phase ultra-high pressure LC (UHPLC) column and analyzed using an Agilent 1290 UHPLC/SCIEX QTrap 5500 LC–MS/MS system in negative electrospray ionization mode. Peak area of individual SCFA was measured against the peak area of corresponding IS to derive the peak area ratio, which was then quantitated against calibration curves of known SCFA concentrations. Stool metabolomics data were reported as concentration of analytes (µmol/g stool).

### Targeted quantitation of immune markers in maternal serum

Targeted quantitation of 92 immune markers was performed in maternal serum across pregnancy (5 timepoints) using the Olink target 96 inflammation panels (Olink Proteomics, Uppsala, Sweden), an aptamer-based multiplex immunoassay. Briefly, immune marker quantitation was performed using Olink Proteomics’ in-house proximity extension assay (PEA) technology. Proteomic data (immune markers) were reported as normalized protein expression (NPX) (normalized based on an arbitrary Log_2_ scale). For samples in which immune markers were below the limit of detection (LOD) of negative controls (*n* = 37) within the assay, values were adjusted to the LOD before proceeding with statistical analyses.

### In vitro mechanistic experiments conducted to validate observations of systemic metabolome perturbations in relation to structural and functional characteristics of the metagenome

For bacterial pure culture experiments, we genotyped and sequence typed three *K. pneumoniae* strains previously isolated from ileal lavage samples of healthy individuals, which showed overall genome relatedness to *K. pneumoniae* strains and different sequence types (Table S1). Protein annotation against the KEGG database (updated 2024-Dec-01) confirmed that all three strains contain the same set of enzymes required for the complete biosynthesis of all three BCAA (Figure S1). *Roseburia intestinalis* DSM 14610^T^ was purchased commercially from the German culture collection of microorganisms (DSMZ). Analysis of the *R. intestinalis* from predicted protein sequences (RefSeq = GCF_000156535.1) against the KEGG database showed a lack of 3-isopropylmalate dehydratase (EC 4.2.1.33), a carbon–oxygen lyase that transforms (2S)-3-isopropylmalate to (2R,3S)-3-isopropylmalate as a precursor for L-leucine biosynthesis. The BCAA biosynthesis experiment was performed according to Supplementary methods using 10 mM glucose as substrate. At specific timepoints (0 and 60 min), culture media was drawn, quenched with equal volume of acetonitrile containing internal standard (IS) (L-tryptophan, indole-D5) prior to in-house LC–MS/MS analyses. The detailed experimental protocol can be found in Supplementary methods section.

A validated in vitro Caco-2 transwell assay protocol was utilized to simulate in vivo systemic absorption of BCAA across colonic lining. Caco-2 cells were passaged and seeded onto 24 well plates (Greiner, product no. 662160) for 21 days (as described in Supplementary methods) to achieve characteristic morphology of differentiated enterocytes exhibiting tight junctions. For absorptive transport (apical to basolateral compartment), solutions (300 µL) containing test compounds (500 µM propranolol (positive control), atenolol (negative control) and individual BCAA) in transport buffer (HBSS containing 50 mM HEPES) were added to the donor apical compartment (A), with “blank” transport buffer (600 µL) in the receiver basolateral compartment (B). At specific timepoints (20, 40, 60, 90 and 120 min), 50 µL of solution would be drawn from the basolateral compartment and diluted with equal volume of ACN containing IS before being analyzed via LC–MS/MS. The detailed LC–MS/MS method for quantitation of analytes are described in Supplementary methods.

### Statistical analyses

Statistical analyses were performed using IBM SPSS 26.0. Descriptive statistics for categorical variables were presented as proportions (%), while continuous variables were presented either as geometric means ± standard deviation (SD) (clinical and metagenomics data) or medians with interquartile range (IQR: 25th −75th quartile) (metabolomics and immune marker proteomics data) as data were non-normally distributed. Differences in categorical and continuous data between groups were analyzed by Chi-square and Mann–Whitney U tests, respectively.

Community-level diversity analyses were performed on maternal and infant gut microbiome profiles. Taxonomic analyses were restricted to bacterial species with a relative abundance ≥ 0.01% to reduce sparsity and ensure robust estimation of community diversity. Alpha-diversity was assessed using the Shannon diversity index. Beta(β)-diversity was assessed using Bray–Curtis dissimilarity, visualized by principal coordinates analysis (PCoA), and statistically evaluated using permutational multivariate analysis of variance (PERMANOVA) to test for differences in community composition between groups across longitudinal timepoints.

Longitudinal analyses were performed using generalized linear mixed models (GLMM) [18] after adjusting for maternal history of allergy during the prenatal period, and maternal antibiotic use during pregnancy/labour and mode of delivery during the postnatal period. These key potential confounders were included in longitudinal comparative analyses given their well-established associations with offspring allergic disease [19, 20] and their known influence on early-life gut microbial colonization and establishment [21, 22]. To account for multiple testing across bacterial taxa (top 150 bacterial taxa with ≥ 0.01% relative abundance), microbial functional gene abundance, and immune markers, *p*-values derived from GLMM were adjusted using Benjamini–Hochberg false discovery rate (FDR) procedure, with an FDR of 10% applied. Longitudinal differences which met this threshold were considered statistically significant; nominal longitudinal differences that did not remain statistically significant after FDR correction are explicitly indicated where relevant.

Maternal plasma metabolites corresponding to perturbed functional gene pathways identified in the third trimester were compared between groups using Mann–Whitney U tests. To account for multiple testing in these analyses, *p*-values were similarly adjusted using Benjamini–Hochberg FDR procedure, with an FDR of 10% applied, and all reported metabolite results reflect FDR-adjusted *p*-values.

Unsupervised factor analysis was conducted on immune markers identified as nominally differentially abundant between groups using a longitudinal screening threshold. Analyses were performed in IBM SPSS Statistics version 31 using principal components extraction based on a correlation matrix, with an eigenvalue threshold > 1 for factor retention. The maximum number of iterations for convergence was set to 25, and Varimax rotation was applied to improve factor interpretability.

For in vitro bacterial experiments, t-tests with FDR corrected *p-*value by the Benjamini–Hochberg method (*q*-value) were performed using GraphPad Prism 7 to compare BCAA levels between timepoints (0 and 60 min). Differences in levels of BCAA between timepoints were statistically significant if the FDR threshold was below 0.05 (*q* < 0.05).

Pathway analyses were performed using generalized structural equation modelling (GSEM) in STATA v18 (StataCorp, College Station, TX) to evaluate hypothesized sequential mediation pathways linking maternal gut microbiome and metabolomic features to infant gut microbiome and AD outcome, with maternal immune markers specified as intermediate mediators. Candidate variables were primarily restricted to features identified as longitudinally significant in prior differential abundance analyses (FDR-adjusted *p* < 0.05); in addition, IL-4 and MMP-1 were included a priori based on established biological relevance to allergic inflammation and immune responses [23–25]. The AD outcome was modelled using ordered logistic regression, while continuous microbial, metabolome and immune marker variables were modelled assuming a Gaussian family. Models were fitted using a robust maximum likelihood estimation, with pairwise deletion applied for missing data (< 5%) and a maximum of 100 iterations allowed for convergence. Omics layers were modelled independently and integrated sequentially (maternal gut microbiome, systemic metabolome, followed by immune markers), with all theoretically plausible direct and indirect paths prespecified. Model refinement was conducted within a confirmatory framework, retaining only paths that were statistically significant after Bonferroni correction (Wald test, *p* < 0.05; controlling for 28 prespecified paths) and that improved model fit (ΔAIC < 2; ΔBIC < 0). Following identification of the final model, maternal-prenatal exposures were included as covariates to evaluate their direct and indirect effects on the retained mediation pathways. Statistical inference at the modelling stage was therefore restricted to a prespecified set of confirmatory hypotheses, with Bonferroni correction applied to control the family-wise error rate.

## Results

In this study, we sought to deconvolute both the (1) maternal-prenatal factors associated with the gut microbiome-systemic metabolome-immune environment during pregnancy and (2) offspring gut microbiome-metabolome signature during early-life in the context of offspring AD. We sequentially investigated and compared the maternal gut microbiome, plasma metabolome and immunological signatures between the two groups, before performing multi-omics integration to uncover their association with maternal clinical and modifiable risk factors (e.g. diet) of offspring AD. Subsequently, the impact of maternal biological factors on the early establishment and maturation of infants’ gut microbiome and metabolome was elucidated to deduce potential prenatal influences leading to offspring AD development.

### Participant demographics and clinical characteristics

Given the nested case–control study design, no significant differences were observed between the control and AD groups in maternal demographics, clinical characteristics and pregnancy factors, or offspring demographics and clinical characteristics (Table 1).

**Table 1:** Comparison of maternal and offspring factors (demographics and clinical characteristics) between mother–offspring dyads from control (*n* = 64) and AD (*n* = 64) group

	**Control (*n* = 64)**		**AD (*n* = 64)**	
	n	%	n	%
*Maternal Factors*				
*Demographics*				
Maternal age (at recruitment) (years)	30 ± 3		30 ± 3	
Maternal income (> 4000 USD)	50	78.1	49	76.6
Education level (tertiary and above)	11	17.2	8	12.5
Currently working	54	85.7	57	89.1
Residential type:				
1. Condominium	7	11.1	4	6.30
2. Government-subsidized housing	52	82.5	56	87.5
3. Landed property	3	4.80	3	4.70
4. Other	1	1.60	1	1.60
*Clinical characteristics*				
Preconception Body Mass Index (BMI; kg/m^2^)	22.9 ± 4.01		22.4 ± 3.67	
Gestational weight gain (first-third trimester)	9.35 ± 3.79		10.4 ± 3.47	
Overweight (BMI ≥ 23)	22	34.9	21	32.8
Obese (BMI ≥ 27.5)	9	14.3	7	10.9
Maternal history of any allergy disorder	31	48.4	38	59.4
Maternal asthma	3	9.70	2	5.30
Maternal eczema	13	20.3	18	28.1
Maternal rhinitis	25	39.1	31	49.2
Any long-term illness (chronic disease)	5	7.90	11	17.2
*Pregnancy factors*				
Probiotics consumed during pregnancy	10	15.6	12	20.0
Antibiotics consumed during pregnancy	16	27.1	15	24.6
Antibiotics administered during labour	23	39.0	23	38.3
Self-reported stress experienced during pregnancy:				
1. None	4	6.30	9	14.1
2. Slightly	28	44.4	22	34.4
3. Moderately	17	27.0	24	37.5
4. A lot	12	19.0	9	14.1
5. Extremely	2	3.20	0	0
*Dietary factors (daily caloric intake)*^1^				
Pre-conception	2,014 ± 709.7		1,991 ± 644.9	
Third trimester (34–36 weeks) *	**2,482 ± 959.0**		**2,707 ± 781.0**	
*Offspring factors*				
Preterm birth (< 37 weeks gestation)	3	4.69	4	6.25
Born by caesarean delivery	16	25.0	19	29.7
Sex (male)	34	53.1	38	59.4
Undergone phototherapy for neonatal jaundice	23	35.9	29	45.3
Presence of biological siblings	21	32.8	24	37.5
Owns a pet	8	12.5	13	20.3
Offspring ethnicity:				
1. Chinese	50	78.1	53	82.8
2. Malay	11	17.2	9	14.1
3. Indian	1	1.60	0	0
4. Mixed	2	3.10	2	3.10
Feeding history (up to 6 months of age):				
1. Exclusive breastfeeding^2^	31	48.4	36	56.3
2. Breastfeeding and formula	6	9.38	8	12.5
3. Exclusive formula	0	0	2	3.13
AD severity score:				
SCORAD at 6 months	NA		10.6 ± 9.22	
SCORAD at 18 months	NA		7.10 ± 8.97	

Data are provided in mean ± SD or n (%) for continuous and categorical data respectively. Bolded variables with * represent statistically significant differences between groups (*p* < 0.05)

^1^Units for caloric intake per day is kcal/day

^2^Exclusive breastfeeding denotes the provision of breast milk only, without formula, for up to 6 months of age; water intake permitted

Additional comparative analyses were performed at each postnatal timepoint to ensure that missing postnatal stool samples did not affect the distribution of offspring demographics and clinical characteristics between groups and introduce confounding effects. No significant differences were found between groups for all demographic and clinical characteristics across all postnatal timepoints except for month 3, where infants in the AD group were more likely to have mothers with positive history of allergy compared to those in the control group (Table S2).

Maternal daily caloric intake did not differ significantly between groups during the pre-conception period. However, mothers in the AD group had significantly higher daily caloric intake in the third trimester as compared to those in the control group (*p* = 0.047; Table 1). Analyses of individual nutrient and food group intakes, normalized by daily caloric intake, in the third trimester revealed no significant differences between groups (Table S3).

### Prenatal gut microbiome and systemic metabolome-immunological signature influences offspring susceptibility to AD development

#### Maternal structural and functional gut microbiome influences the systemic metabolome in relation to offspring AD outcomes

Shannon diversity did not differ between mothers in the AD and control group at any prenatal timepoint (Figure S2A). Consistently, Bray–Curtis β-diversity showed no significant differences in overall community composition, with no group separation observed by PCoA and no significant group effects detected by PERMANOVA (Figure S2B).

**Fig. 2:**
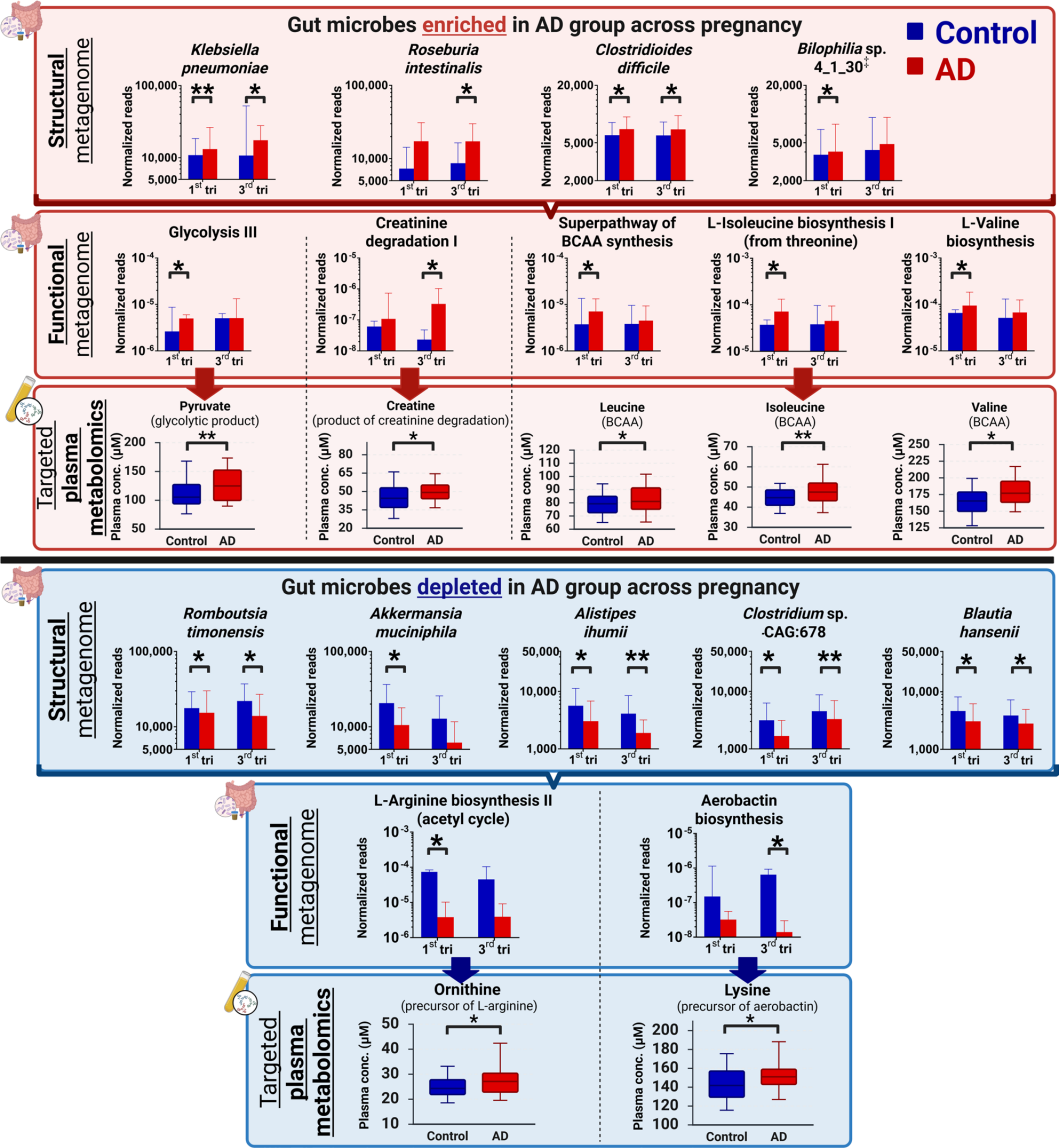
Structural and functional metagenomics analyzed in tandem with targeted plasma metabolomics to interrogate significant gut microbiome-systemic metabolome perturbations between groups. We identified gut bacteria, and their associated functional gene carriage, which were significantly enriched (red panels – top and middle) or depleted (blue panels – top and middle) in the AD group across pregnancy based on FDR-adjusted longitudinal *p*-values (< 0.05), after adjusting for maternal history of allergy. The symbol ‡ denotes taxa showing nominal longitudinal significance that did not meet the FDR threshold. Targeted plasma metabolomics was performed at the third trimester of pregnancy to quantitate metabolic intermediates within the pre-identified functional gene pathways. The significantly perturbed metabolites corresponded to (1) an accumulation of downstream metabolic products (red panels – bottom) in enriched functional gene carriage and (2) an accumulation of upstream metabolic precursors (blue panels – bottom) in depleted functional gene carriage in the AD group (FDR-adjusted *p*-values < 0.05). Metagenomic data are presented as geometric mean and geometric standard deviation range of normalized reads in log-scale while metabolomics data are presented as median and (interquartile range: 25th – 75th percentile) in µM. * Represents significant difference between groups of *p* < 0.05 at specific pregnancy timepoint; ** Represents significant difference between groups of *p* ≤ 0.01 at specific pregnancy timepoint

From the maternal gut metagenomic taxonomic profile, considering only major abundant bacteria with a minimum of 0.1% relative abundance, fourteen bacteria species were found to be significantly different at either first or third trimester of pregnancy between groups (*p* < 0.05) (Table S4). When compared temporally across pregnancy, normalized read counts of *Klebsiella pneumoniae*, *Roseburia intestinalis* and *Clostridioides difficile* were significantly enriched in mothers of AD infants compared to control group (FDR-adjusted longitudinal *p* < 0.05) whereas *Clostridium* sp. CAG:678*, Romboutsia timonensis*, *Akkermansia muciniphila*, *Blautia hansenii* and *Alistipes ihumii* were depleted (FDR-adjusted longitudinal *p* < 0.05;Fig. 2; Table S4). *Bilophila* sp. 4_1_30 was also found to be longitudinally nominally elevated in mothers from the AD group across, but this difference did not reach statistical significance after FDR adjustment (Table S4).

We then conducted stratified analyses of metabolic pathways associated with these nine perturbed bacterial taxa across pregnancy, using functional annotations from the MetaCyc database [26]. The four bacteria that were enriched in AD (*K. pneumoniae*, *R. intestinalis*, *C. difficile* and *Bilophilia* sp. 4_1_30) contributed to a significantly higher read abundance for several metabolic pathways, namely glycolysis III (from glucose), creatinine degradation I, superpathway of branched-chain amino acid (BCAA) biosynthesis, L-isoleucine biosynthesis I (from threonine) and L-valine biosynthesis) (FDR-adjusted longitudinal *p* < 0.05) (Table S5). In contrast, *Clostridium* sp. CAG:678*, R. timonensis*, *A. muciniphila*, *B. hansenii* and *A. ihumii,* which were depleted in AD group, resulted in a significantly lower read abundance for several metabolic pathways, namely L-arginine biosynthesis II (acetyl cycle) and aerobactin biosynthesis (FDR-adjusted longitudinal *p* < 0.05) (Table S5).

Next, we utilized targeted metabolomics to quantitate specific metabolites in maternal plasma in the third trimester (27–28 weeks) of pregnancy, focusing on metabolites that were constituents of previously identified enriched or depleted functional gene carriage analyses arising from the nine identified bacteria.

We identified significantly higher plasma concentrations of (1) downstream metabolic products from enriched functional gene pathways: leucine, isoleucine and valine (from BCAA biosynthesis super-pathway, L-isoleucine and L-valine biosynthesis), creatine (from creatinine degradation I) and pyruvate (from glycolysis III) (FDR-adjusted *p* < 0.05) and (2) upstream metabolic precursors in depleted functional gene pathways: ornithine (from L-arginine biosynthesis II) and lysine (from aerobactin biosynthesis) (FDR-adjusted *p* < 0.05) in AD group as compared to the control group (Fig. 2 and Table S6). These results suggest the potential for systemic metabolome perturbations to arise from the aberrant maternal-prenatal gut microbial signature (Fig. 2).

#### Mechanistic work to validate systemic metabolome perturbations (e.g. BCAA) arising from gut microbiome signature (*K. pneumoniae* and *R. intestinalis*)

To test the premise that enrichments in specific high abundance bacteria (such as *K. pneumoniae* and *R. intestinalis*) during pregnancy could contribute to elevated systemic circulating levels of gut microbial metabolites (e.g. BCAA), we performed in vitro experiments to investigate (1) whether we could measure BCAA in the supernatant of the implicated faecal bacteria, which could suggest similar release into the intraluminal colonic compartment; and (2) whether the secreted metabolites are systemically absorbed from within the intraluminal colonic compartment into the systemic circulation.

BCAA measurements of resting cells spiked with glucose revealed that *K. pneumoniae* and *R. intestinalis* synthesized and secreted specific BCAA into culture media (Figure S3A). While *K. pneumoniae* produced and secreted all three BCAA, *R. intestinalis* synthesized only isoleucine and valine (Figure S3A), consistent with their individually annotated functional metagenome.

Next, we investigated the flux of these metabolites across an in vitro Caco-2 monolayer to simulate systemic absorption of gut microbial-derived metabolites from the intestinal lumen across the colonic lining. Flux of high and low-permeability markers (propranolol and atenolol respectively) were used as positive and negative controls respectively to validate Caco-2 transwell assay functionality (Figure S3B—right). Incubation of BCAA within apical transwell compartment demonstrated time-dependent flux of all three BCAA from the apical compartment to the basolateral compartment in linear fashion over 120 min (Figure S3B—left).

#### Maternal immunological environment in the AD group was skewed towards T-helper 2 (T_H_2) and pro-inflammatory immune markers

Next, we sought to interrogate the maternal immunological environment during pregnancy and its potential role in the development of offspring AD.

**Fig. 3:**
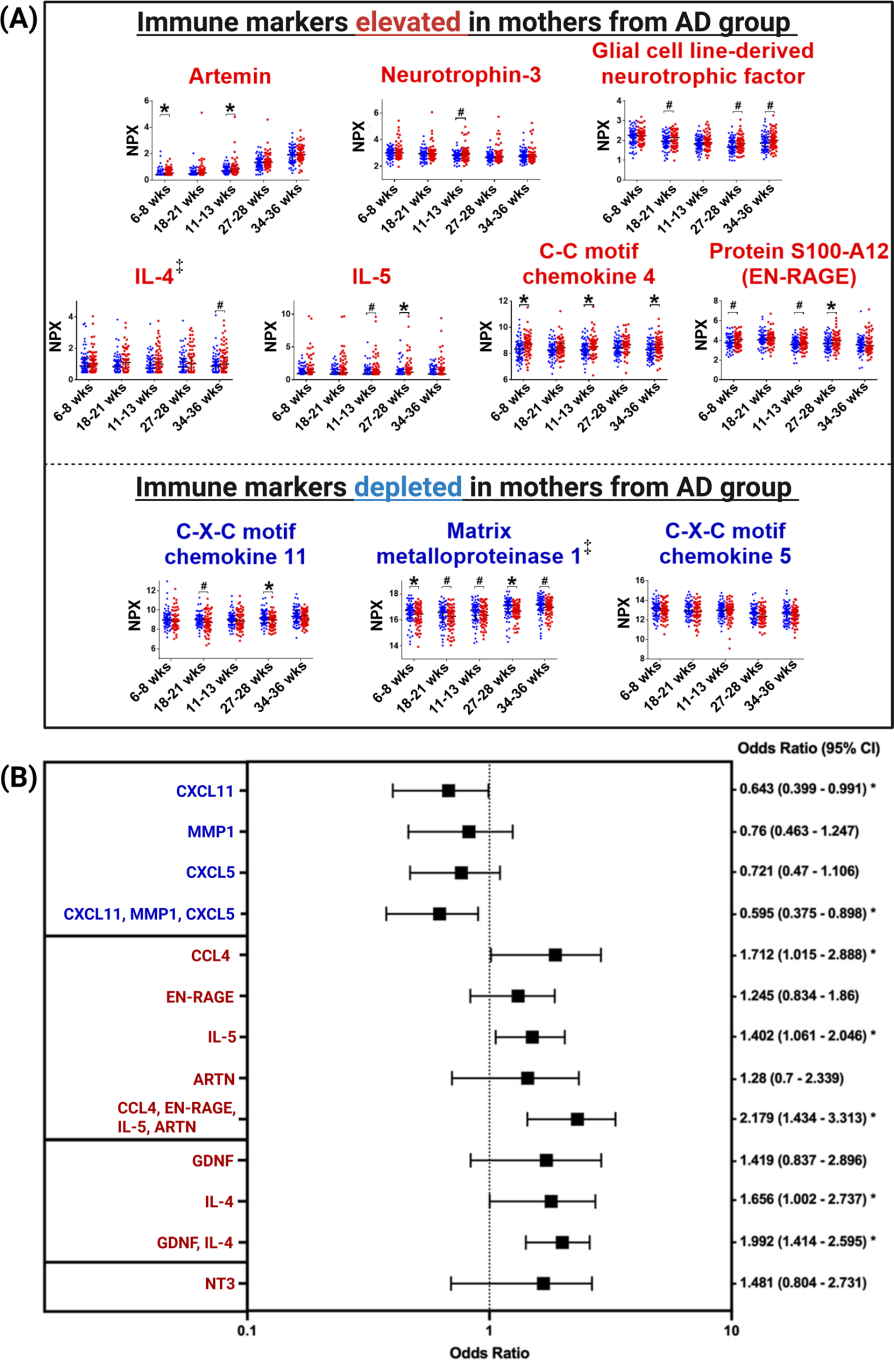
Maternal immune environment in the leadup to offspring AD. **A** Maternal immune markers shown to be elevated (upper panel) or lowered (lower panel) in the AD group across five pregnancy timepoints based on FDR-adjusted longitudinal *p*-values (< 0.1). Group differences at individual timepoints were assessed using Mann–Whitney U tests, while longitudinal trajectories were evaluated using generalized linear mixed models adjusted for maternal history of allergy. The symbol ‡ denotes immune markers with nominal longitudinal significance that did not meet the FDR threshold. **B** Forest plot depicting the odds ratio (OR) and 95% confidence intervals (CI) from logistic regression, assessing the relationship between individual immune markers, four groups of immune markers (clustered by factor analysis) and offspring AD outcomes. OR > 1 denotes the increased offspring AD odds, while an OR < 1 denotes the reduced offspring AD odds. *denotes significance *p* < 0.05. ^#^denotes 0.05 < *p* < 0.1

We found that the maternal immunological signature in the AD group was characterized by higher levels of T-helper 2 (T_H_2) associated cytokine (IL-5), neurotrophins (artemin (ARTN), glial cell line-derived neurotrophic factor (GDNF) and neurotrophin-3 (NT3)) and pro-inflammatory innate immune markers (C–C motif chemokine 4 (CCL4) and protein S100-A12 (EN-RAGE)) and lower levels of T-helper 1 (T_H_1) associated inflammation marker (C-X-C motif chemokine 11 (CXCL11)) and other inflammation markers (C-X-C motif chemokine 5 (CXCL5)) across pregnancy as compared to the control group (FDR-adjusted longitudinal *p* < 0.1; Fig. 3A; Table S7). IL-4 (another important T_H_2 cytokine) was also longitudinally nominally elevated, while matrix metalloproteinase 1 (MMP-1) was found to be longitudinally nominally depleted in the AD group across pregnancy, although these differences were not statistically significant after FDR-adjustment (Table S7).

We performed unsupervised factor analysis to explore plausible statistical associations and biological relevance of these 10 differentially expressed immune markers in relation to offspring AD predisposition. These analyses identified four distinct clusters: (1) CXCL11, MMP-1 and CXCL5; (2) CCL4, EN-RAGE, IL-5 and ARTN; (3) GDNF and IL-4; and (4) NT3. Notably, the combination of CXCL11, MMP-1 and CXCL5 further reduced the odds of offspring AD development compared to each individual immune marker. Conversely, the combinations of (1) CCL4, EN-RAGE, IL-5, ARTN and (2) GDNF, IL-4 further increased odds of offspring AD development compared to their respective individual markers (Fig. 3B).

#### Multi-omic integration of prenatal gut microbiome, systemic metabolome and immunological signature reveals a distinct prenatal-maternal signature in relation to offspring AD outcomes

**Fig. 4:**
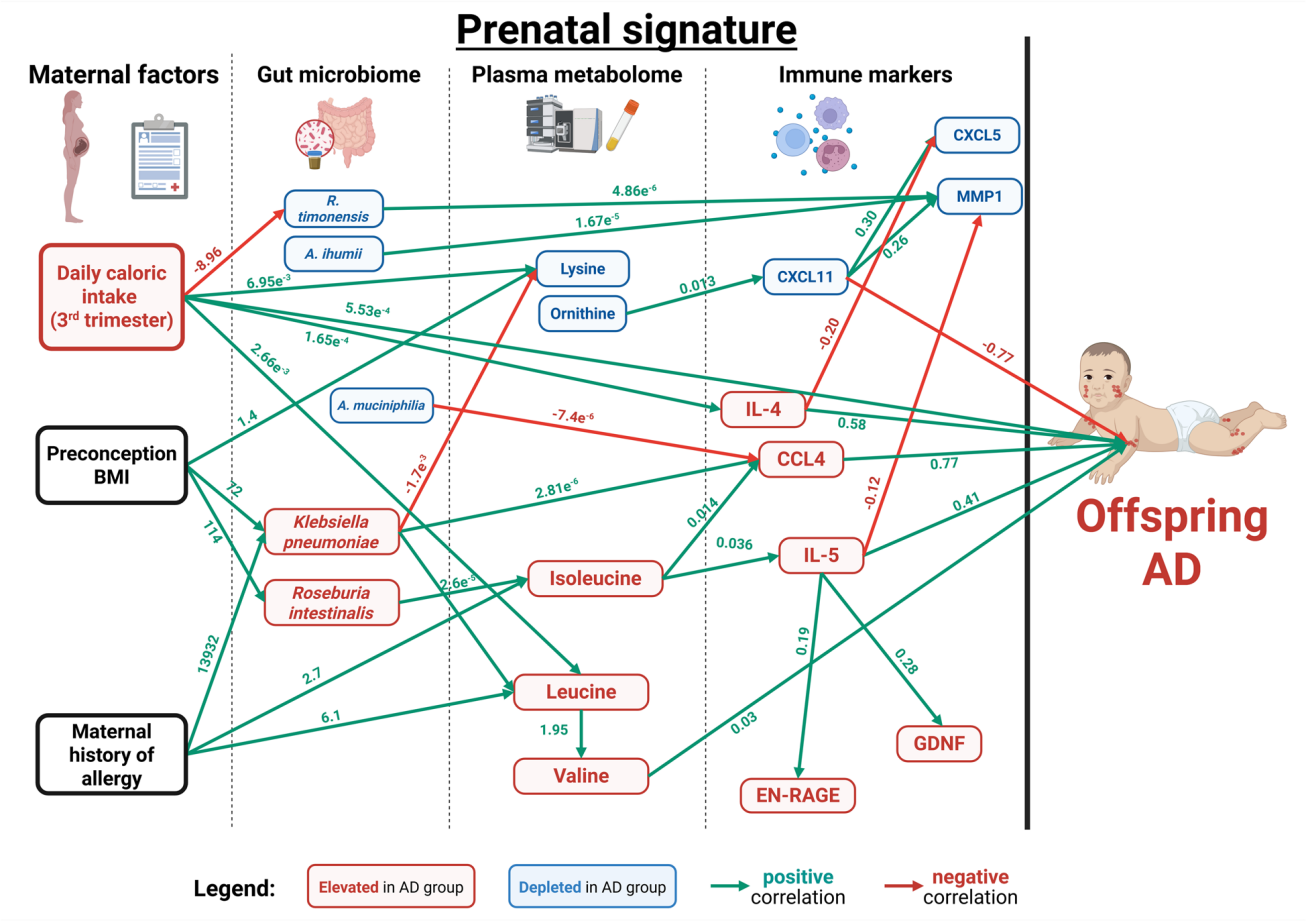
Generalized structural equation model for associations between maternal factors, gut microbiome, circulating metabolites and immune markers and infant AD outcome. Only significant paths and their estimates, after adjusting for maternal history of allergy, were shown in the model at adj *p* < 0.05. Green and red lines indicate the significant positive and negative associations, respectively, between variables. Green or red numbers represent positive or negative correlation coefficients respectively between variables and reflect the associations between variables following the direction of the arrow

We performed a supervised pathway analysis using generalized structural equation model (GSEM) to identify sequential statistical relationships between the maternal clinical characteristics and multi-omics variables (metagenomics, metabolomics followed by immune markers) in relation to offspring AD outcome (Fig. 4). We identified four important paths: (1) Maternal preconception BMI and history of allergy were independent factors which were positively associated with *K. pneumoniae* enrichment, elevated plasma leucine and valine and increased odds of offspring AD outcome; (2) Maternal preconception BMI was positively associated with *R. intestinalis*, increased plasma isoleucine, CCL4 and IL-5 and increased odds of offspring AD outcome; (3) Maternal daily caloric intake (at third trimester) was positively associated with plasma leucine, lysine and IL-4, and increased odds of offspring AD outcome and (4) T_H_2 cytokine (IL-4, IL-5) and T_H_1 chemokine, CXCL11, are immunological hubs that directly influence odds of offspring AD outcomes (adj *p* < 0.05). Other notable associations include *A. muciniphilia* which was negatively associated with CCL4, while *R. timonensis* and *A. ihummi* were positively associated with MMP1. T_H_2 cytokines (IL-4, IL-5) were negatively associated with anti-inflammatory markers MMP1 and CXCL5, while positively associated with other pro-inflammatory markers EN-RAGE and GDNF (adj *p* < 0.05).

### Early-life gut microbiome-metabolome perturbations precede offspring AD development

Shannon diversity was similar between healthy and AD infants across postnatal time points, except at month 3, when it was higher in healthy infants (Figure S2A). Bray–Curtis β-diversity revealed no significant differences in community composition at any postnatal timepoint, with no group separation on PCoA and no significant effects detected by PERMANOVA (Figure S2B).

**Fig. 5:**
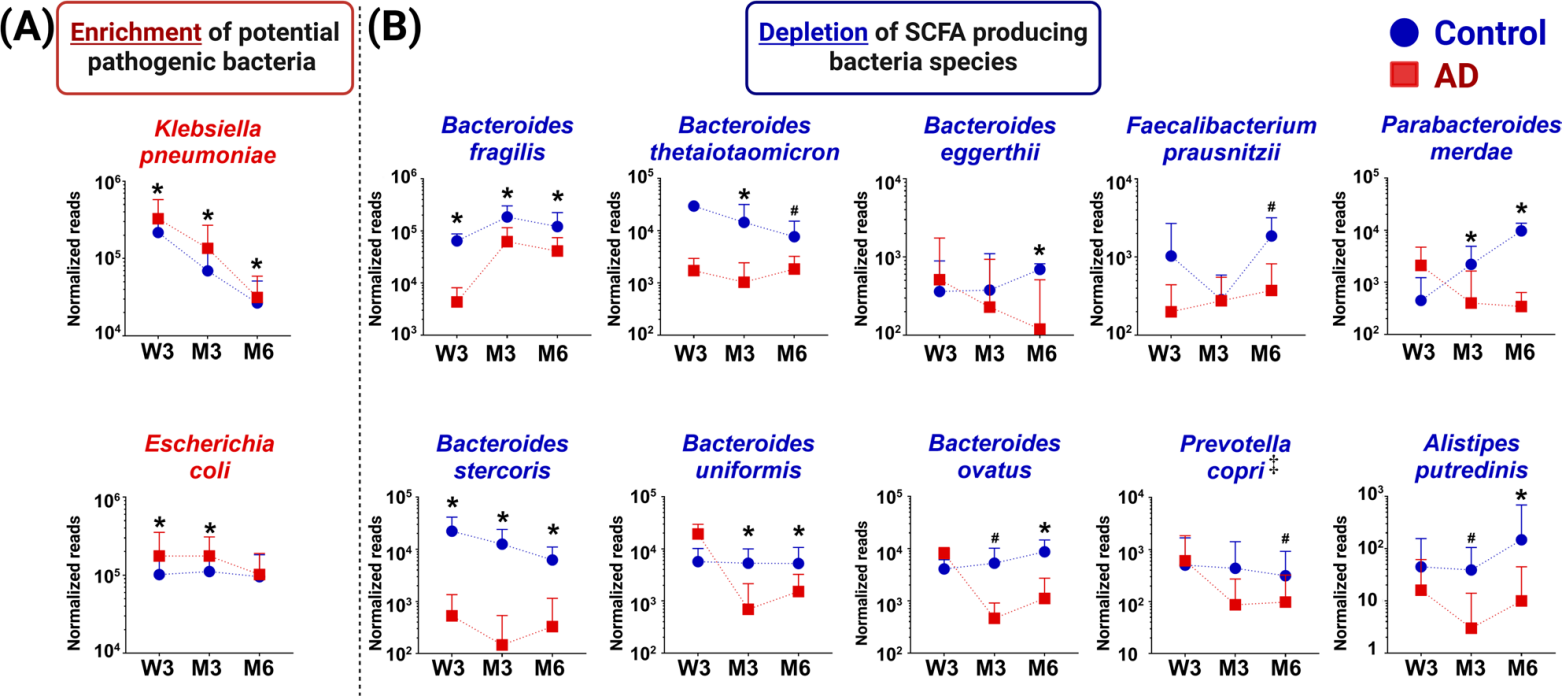
Longitudinal gut microbiome perturbations of AD infants during early-life. Gut bacteria (with minimum 0.1% relative abundance) which were demonstrated to be either significantly **A** enriched or **B** depleted in AD infants temporally during early life based on FDR-adjusted longitudinal *p*-values (< 0.05) after adjusting for maternal antibiotic use during pregnancy/labour and mode of delivery. The symbol ‡ denotes taxa showing nominal longitudinal significance that did not meet the FDR threshold. Data are presented as geometric mean and geometric standard deviation range of normalized reads in log-scale. Comparison of normalized reads between control and AD groups at individual timepoints are shown in Table S7. *Represents significant difference between groups at *p* < 0.05 at specific timepoint. ^#^denotes 0.05 < *p* < 0.1

Throughout infancy from week 3 to month 6, normalized read counts of several potentially pathogenic members of *Enterobacteriaceae* family (*Klebsiella pneumoniae*, *Escherichia coli)* were enriched in AD compared to control infants (adj *p* < 0.05) (FDR-adjusted longitudinal *p* < 0.05; Fig. 5A; Table S8). In contrast, short chain fatty acid (SCFA)-producers from genus Bacteroides (*Bacteroides fragilis*, *Bacteroides stercoris*, *Bacteroides uniformis*, *Bacteroides thetaiotaomicron*, *Bacteroides eggerthii* and *Bacteroides ovatus*) and others such as *Alistipes putredinis*, *Parabacteroides merdae* and *Faecalibacterium prausnitzii* were depleted in the AD compared to the control group (FDR-adjusted longitudinal *p* < 0.05; Fig. 5B; Table S8). *Prevotella copri* (an important SCFA producer) was also longitudinally nominally depleted in AD infants from week 3 to month 6, but this difference did not reach statistical significance after FDR adjustment (Table S8). Consistent with the depleted SCFA producing microbes, there were significantly decreased levels of stool SCFA, namely, isovaleric acid, 2-methylbutyric acid and isobutyric acid, at three months of age in AD infants as compared to control infants (Table S8).

### Maternal biological signatures (gut microbiome and immune environment) affect the establishment of gut microbes in offspring, which were linked to AD development

**Fig. 6:**
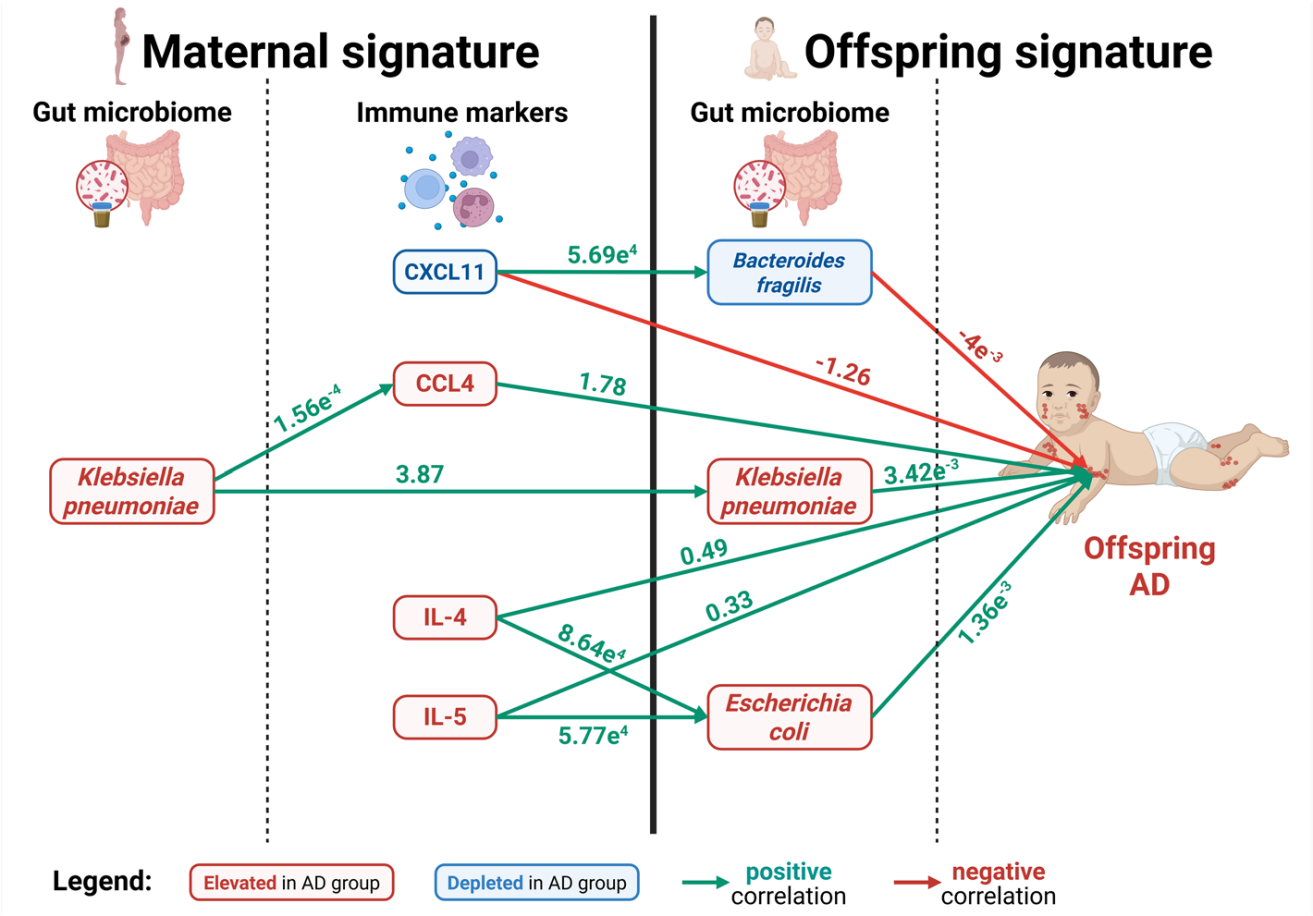
Generalized structural equation model to study associations between maternal gut microbiota and immune markers at the third trimester, infant gut microbiome at week 3 and infant AD outcome. Only significant paths and their estimates were shown in the model at *p* < 0.05. Green or red numbers represent positive or negative correlation coefficients respectively between variables and reflect the associations between variables following the direction of the arrow

To interrogate the influence of maternal biological signature during pregnancy on the establishment of the infant early-life microbiome, we expanded the previously developed GSEM (Fig. 4) to incorporate both prenatal and postnatal periods. We included only samples (18 Controls vs 8 AD) with complete maternal gut microbiome, plasma metabolome and immune marker data during pregnancy (third trimester) and infant stool microbiome at week 3 (Fig. 6). We observed: (1) a strong positive association between maternal and infant gut *K. pneumoniae*; (2) positive associations between maternal IL-4 and IL-5 and infant gut *E. coli*; (3) positive associations between maternal CXCL11 and infant *B. fragilis,* which were both associated with decreased odds of offspring AD and (4) infant gut *K. pneumoniae* and *E. coli* were associated with increased odds of offspring AD outcome while *B. fragilis* was associated with decreased offspring AD odds.

## Discussion

The “Developmental Origins of Health and Disease” (DOHaD) concept postulates that specific exposures during critical junctures of early-life development (from the prenatal period to early-life) significantly alter an individual’s long-term health [27]. Our study has pioneered the utilization of a longitudinal multi-omics approach to uncover a maternal biological signature linking perturbations extending throughout pregnancy to the offspring’s early-life, culminating in an elevated risk of offspring AD development.

While several studies have provided empirical evidence alluding to the potential protective or detrimental effect of the maternal gut microbiome-systemic metabolome in relation to offspring allergic disease outcomes [28, 29], none have comprehensively linked these perturbations to underlying immunological mechanisms. We hypothesized that the collective maternal gut microbiome-systemic metabolome signature may be associated with a pro-inflammatory and T_H_2-skewed immune milieu during pregnancy, driving the consequent risk of offspring AD development.

In the AD group, we discovered a maternal gut microbial signature during pregnancy which was characterized by enriched (*K. pneumoniae*, *R. intestinalis*, *C. difficile* and *Bilophilia* sp. 4_1_30) and depleted (*Clostridium* sp. CAG:678*, R. timonensis*, *A. muciniphila*, *B. hansenii* and *A. ihumii*) gut microbes (Fig. 2). Although these specific microbes have not been directly linked to offspring AD development in the prenatal context, enrichment of well-known pathogens (*K. pneumoniae* and *C. difficile*) have been shown to synergistically induce pro-inflammatory responses in the intestine [30]. In contrast, the depletion of *A. muciniphilia*, known for its anti-inflammatory effects on macrophages [31], could impair protective effects on the intestinal lining [32] exacerbating inflammation and compromising gut health.

In vitro pure bacterial culture and Caco-2 cell culture experiments (Figure S3) validated the systemic role elicited by the maternal gut microbiome (specifically *K. pneumoniae* and *R. intestinalis*) as key modulators of the systemic metabolome (BCAA), during pregnancy. The comparable protein intake between groups (Table S3) supports a microbial rather than dietary origin for these metabolic perturbations. Elevated BCAA levels overtly stimulate peripheral blood mononuclear cells, inducing oxidative stress and a pro-inflammatory phenotype [33] while other metabolites, such as creatine and ornithine, skew immune response towards a T_H_2 phenotype [34, 35]. Notably, maternal plasma propionic acid, an important gut microbial-derived immunomodulatory SCFA, was also significantly reduced during the third trimester (data not shown) in the AD group, despite no corresponding alterations to its related functional gene expressions.

Analyses of the prenatal immune environment, adjusted for maternal allergic history, revealed distinct clusters of maternal immune markers of mothers associated with offspring AD outcomes (Fig. 3B). These clusters showed a bias towards persistently elevated T_H_2 cytokines, neurotrophic factors and pro-inflammatory innate immune markers (IL-5, CCL4, EN-RAGE and ARTN) and decreased T_H_1 (CXCL11) and immune modulating (CXCL5) chemokines (Fig. 3B). IL-5 plays an integral role in eosinophil hematopoiesis, recruitment and survival [36] and has been shown to cross the maternal placenta and prime the fetal immune system in utero leading to fetal eosinophilia [37]. Novel innate pro-inflammatory immune markers, such as CCL4 and EN-RAGE, may independently trigger allergic disease onset through eosinophilic recruitment [38] and neutrophilic inflammatory response [39], respectively, while neurotrophic factors (such as ARTN) may contribute to allergic disease development through activation of inflammatory signaling pathways [40] and sustenance of eosinophilic response [41]. In contrast, CXCL11 promotes T_H_1-associated immunological responses [42] and lower levels during infants’ infancy have been shown to increase odds of allergic disease outcomes in later-life [43], while increased CXCL5 has been shown to modulate excessive inflammatory responses [44]. While the interplay of these pro- and anti-inflammatory immune markers offer promising insight to the prenatal immune environment linked to offspring allergic disease development, further mechanistic studies are necessary to interrogate their specific immunological roles.

By incorporating maternal factors and altered prenatal omics variables sequentially, we propose a holistic maternal-prenatal signature that undergirds offspring AD development (Fig. 4). Firstly, we showed that highly abundant enriched gut microbes (*K. pneumoniae* and *R. intestinalis*) were positively associated with systemic BCAA and pro-inflammatory immune markers (IL-5 and CCL4), which increased offspring AD odds (Fig. 4). Secondly, our GSEM also identified the direct influence of T_H_2- (positive correlation) and T_H_1-associated (negative correlation) immune markers on offspring AD outcomes. These findings align with those of other studies showing that a maternal immune environment overtly skewed towards T_H_2 rather than T_H_1 immune markers during pregnancy is associated with an increased risk of offspring allergic disease outcomes [45]. Thirdly, we identified plausible biological mechanisms through which a modifiable maternal-prenatal factor, daily caloric intake during pregnancy, may augment offspring susceptibility to AD. Notably, during pregnancy, mothers of AD infants consumed higher daily caloric intake than those from the control group (Table 1), which exceeded the recommended level of approximately 2500 kcal/day [46]. Given that mothers in the AD group consumed more calories daily, reflecting a generalized increase in nutrient and food groups rather than a specific rise in any one component (Table S3), future research is necessary to clarify how compositional changes in various food group components contribute to the total caloric intake. This may help distill the influence of dietary patterns on the maternal biological signature, ultimately informing more targeted prenatal dietary recommendations for the prevention of offspring atopy.

Based on the integrated analyses, our study found that the observed maternal gut microbiome dysbiosis and pro-inflammatory immunological milieu may serve as early antecedent factors contributing towards offspring allergic disease predisposition. In AD infants, we identified sustained enrichments in potentially pathogenic *Enterobacteriaceae* (*K. pneumoniae* and *E. coli*) and depletions in beneficial SCFA producers (*Bacteroides spp., P. copri, F. prausznitzii, P. merdae, A. putredinis*) (Fig. 5). The findings of enriched *K. pneumoniae*, *E. coli* and depleted *B. fragilis* specifically in the first 100 days of life corroborate similar observations from our previous study which was derived from an independent multiethnic GUSTO (Growing Up in Singapore Towards healthy Outcomes) cohort in Singapore [12] and are consistent with other reports [47]. Notably, a low abundance of *B. fragilis* in early life has also been associated with Caesarean delivery, a known risk factor for eczema development [48]. Albeit not significantly different between groups, a higher proportion of AD infants underwent Caesarean delivery (Table S2). Members of the *Bacteroides* genera have been identified as biomarkers of infants born vaginally and breastfed [49] and can metabolize human milk oligosaccharides into SCFA [50]. Among other depleted bacteria, *F. prausznitzii* is an important butyrate producing commensal bacteria [51] while *P. copri* and *A. putredinis* are known SCFA producers [52]. Congruently, the stool of AD infants contained significantly decreased levels of SCFA (isovalerate, isobutyrate and 2-methylbutyrate) as compared to healthy infants. Mechanistic studies have demonstrated that these SCFA perform important immunomodulatory functions by inhibiting the NF-κB pathway and dampening pro-inflammatory cytokine expression [53], thereby promoting and maintaining intestinal epithelial integrity [53], which may reduce the risk of allergic disease development.

Collectively, given the strong association between the maternal factors and offspring atopic outcomes [54, 55], we hypothesized that the impaired gut microbiome establishment in AD infants may result from both dysregulated maternal-offspring transfer of specific enriched maternal gut bacteria and the maternal immunological milieu across pregnancy [56]. We identified a strong positive statistical correlation between maternal and offspring *K. pneumoniae* (Fig. 6), supporting studies that suggest the gut microbiome of atopic offspring may be colonized by specific maternally derived *Enterobacteriaceae* species [57]. As vertical transmission of maternal “heirloom” microbes are responsible for establishing offspring early-life gut microbiome [58], our findings allude to maternally enriched *Klebsiella* (and not *Roseburia*) as the putative key culprit for offspring’s early microbial “seeding”, which is ultimately linked to AD development. However, due to the smaller postnatal sample size, our study cannot rule out the contribution of other clinical factors, such as mode of delivery or intrapartum antibiotics prophylaxis, on maternal-offspring *Klebsiella* transfer.

Secondly, maternally perturbed T_H_2 (IL-4 and IL-5) and T_H_1-associated (CXCL11) immune markers were linked to an enrichment of offspring *E. coli* and depletion of *B. fragilis* (Fig. 6), suggesting that the impaired early-life gut microbial signature in AD offspring may be influenced by the dysregulated maternal immunological environment. We postulate that the maternal immune system could mediate offspring early life gut microbiome establishment by (1) modulating the maternal-prenatal microbiome and its subsequent transfer to offspring and (2) programming the offspring’s immunological environment in utero [59]. The neonatal immune system at birth is intricately linked to the development of the early-life gut microbiome [60], with intestinal immunity and inflammation regulating the composition and maturation of the gut structural community during early-life [61]. Taken together, our findings indicate that the maternal-prenatal immune environment plays an important role in shaping the neonatal gut microbial environment establishment, which in turn affects allergic disease predisposition.

Firstly, we acknowledge the limitations of inferring differences in microbial metabolic pathway activity between groups solely from stool metagenomics. To address this, we measured specific circulating plasma metabolites (e.g. BCAA) to validate and support these functional predictions. Secondly, caution should be taken in interpreting some results observed in the smaller sub-samples. Although the distribution of clinical characteristics did not differ significantly between groups postnatally, we cannot rule out the collective effect of all postnatal and environmental factors on the observed compromised early-life gut microbial signature in AD infants. Thirdly, given the complex interplay between the maternal gut microbiome, systemic metabolome and immunological milieu and the offspring gut microbiome-metabolome, definitive causality between these variables cannot be established and the observed relationships, while significant, may be influenced by other unmeasured factors and interact in a bidirectional manner. Nevertheless, the longitudinal multi-omics nature of our study enabled us to integrate individual omic variables holistically to deconvolute the temporal biological signature during pregnancy that underlie offspring gut microbial dysbiosis and subsequent AD development. Finally, the inherent limitations of our nested case–control study design preclude the precise identification of maternal clinical factors driving this biological signature. Future large-scale, longitudinal and intervention studies with comprehensive clinical profiling will be essential to disentangle these complex interactions to identify specific maternal clinical factors that shape this biological signature.

## Conclusions

Our study provides novel insight into the complex interplay between maternal gut microbiome dysbiosis, systemic metabolome perturbations, and their association with a T_H_2-biased and pro-inflammatory immune environment during pregnancy – factors that increase offspring susceptibility to AD. Moreover, this distinct maternal-prenatal biological signature shapes the consequent development of a compromised early-life gut microbiome-metabolome profile in offspring who subsequently develop AD, highlighting new pathways that link maternal health during pregnancy to offspring atopic outcomes. Our findings emphasize the prenatal period as a critical window of opportunity for potential clinical interventions, such as maternal dietary modifications [62], to mitigate risk of offspring atopy.

## Supplementary Information

Additional file 1: Table S1. Genomic information and phylogenetic identity of the *Klebsiella pneumoniae* strains. Table S2. Comparison of demographic and clinical characteristics between infants from control and AD groups during various timepoints during early-life. Table S3. Comparison of nutrient and food group data between mothers from control and AD groups at the third trimester of pregnancy. Table S4. Differential bacteria between mothers from control and AD groups which were identified at both first and third trimester of pregnancy. Table S5. Enriched or depleted functional gene carriage between mothers from control and AD groups at both first and third trimesters of pregnancy. Table S6. Targeted plasma metabolomics of specific metabolites identified from functional genomic pathways of differential gut bacteria between groups, were performed in mothers at third trimester (27-28 weeks) of pregnancy. Table S7. Comparison of maternal-prenatal immune markers between groups across five pregnancy timepoints. Table S8. Differential gut bacteria and perturbed stool metabolites between control and AD infants during offspring early life. Figure S1. KEGG biosynthesis pathways for valine, leucine and isoleucine. Figure S2. Community-level gut microbiome diversity across prenatal and postnatal timepoints. Figure S3. In vitro mechanistic experiments demonstrating (A) bacterial mediated synthesis of gut microbial-derived metabolites and (B) flux of these across an in vitro cellular colonic model.

## Data Availability

Shotgun metagenomic sequencing data have been deposited into NCBI, under BioProject number PRJNA1273620 (https://www.ncbi.nlm.nih.gov/bioproject/PRJNA1273620/) [63]. The raw sequencing data for the Klebsiella pneumoniae strains WHN643, WHN880 and WHN1212 have been deposited into NCBI under the BioProject number PRJNA1157478 with SRA numbers SRR31838646 to SRR31838648 and Biosample accession numbers SAMN45967172 to SAMN45967174. Roseburia intestinalis DSM 14610T predicted protein sequences (RefSeq no. GCF_000156535.1) were downloaded from the NCBI dataset. Other clinical data from the Singapore PREconception Study of long-Term maternal and child Outcomes (S-PRESTO) cohort study are not publicly available due to the multi-institutional cohort data governance. The S-PRESTO Executive Committee reviews the data access request and approves the distribution of data.

## Abbreviations

AD: Atopic dermatitis
ARTN: Artemin
BCAA: Branched-chain amino acids
BMI: Body mass index
CCL4: C–C motif chemokine 4
CXCL5: C-X-C motif chemokine 5
CXCL11: C-X-C motif chemokine 11
DOHaD: Developmental Origins of Health and Disease
EN-RAGE: Protein S100-A12
FFQ: Food frequency questionnaire
GC–MS/MS: Gas chromatography tandem mass spectrometry
GDNF: Glial cell line-derived neurotrophic factor
GSEM: Generalized structural equation model
GUSTO: Growing Up in Singapore Towards healthy Outcomes
IL-4: Interleukin-4
IL-5: Interleukin-5
IS: Internal standard
IUCD: Intrauterine contraceptive device
LC–MS/MS: Liquid chromatography tandem mass spectrometry
LCA: Lowest-common-ancestor
LOD: Limit of detection
MMP-1: Matrix metalloprotease 1
NPX: Normalized Protein eXpression
NT-3: Neurotrophin 3
SCFA: Short chain fatty acids
SCORAD: SCORing Atopic Dermatitis
S-PRESTO: Singapore PREconception Study of Long-Term Maternal and Child Outcomes
T_H_1: T-helper 1
T_H_2: T-helper 2
UHPLC: Ultra high pressure liquid chromatography

## References

[1] Odhiambo JA, Williams HC, Clayton TO, Robertson CF, Asher MI, Group IPTS. Global variations in prevalence of eczema symptoms in children from ISAAC phase three. J Allergy Clin Immunol. 2009;124(6):1251-8. e23. 20004783 10.1016/j.jaci.2009.10.009

[2] Ahn C, Huang W. Clinical presentation of atopic dermatitis. In: Management of atopic dermatitis: methods and challenges. 2017. p. 39–46.

[3] Lee BW, Detzel PR. Treatment of childhood atopic dermatitis and economic burden of illness in Asia Pacific countries. Ann Nutr Metab. 2015;66(Suppl. 1):18–24. 25925337 10.1159/000370221

[4] Boguniewicz M, Leung DY. Atopic dermatitis: a disease of altered skin barrier and immune dysregulation. Immunol Rev. 2011;242(1):233–46. 21682749 10.1111/j.1600-065X.2011.01027.x PMC3122139

[5] Budu-Aggrey A, Kilanowski A, Sobczyk MK, Team aR, Shringarpure SS, Mitchell R, et al. European and multi-ancestry genome-wide association meta-analysis of atopic dermatitis highlights importance of systemic immune regulation. Nat Commun. 2023;14(1):6172. 37794016 10.1038/s41467-023-41180-2 PMC10550990

[6] Gao Y, Nanan R, Macia L, Tan J, Sominsky L, Quinn TP, et al. The maternal gut microbiome during pregnancy and offspring allergy and asthma. J Allergy Clin Immunol. 2021;148(3):669–78. 34310928 10.1016/j.jaci.2021.07.011

[7] Koren O, Goodrich JK, Cullender TC, Spor A, Laitinen K, Bäckhed HK, et al. Host remodeling of the gut microbiome and metabolic changes during pregnancy. Cell. 2012;150(3):470–80. 22863002 10.1016/j.cell.2012.07.008 PMC3505857

[8] Mishra A, Lai GC, Yao LJ, Aung TT, Shental N, Rotter-Maskowitz A, et al. Microbial exposure during early human development primes fetal immune cells. Cell. 2021;184(13):3394-409. e20. 34077752 10.1016/j.cell.2021.04.039 PMC8240556

[9] Loo EXL, Soh SE, Loy SL, Ng S, Tint MT, Chan S-Y, et al. Cohort profile: Singapore preconception study of long-term maternal and child outcomes (S-PRESTO). Eur J Epidemiol. 2021;36:129–42. 33222050 10.1007/s10654-020-00697-2 PMC7116651

[10] Hanifin JM. Diagnostic features of atopic dermatitis. Acta Derm Venereol(Stockh). 1980;92:236.

[11] Lim SX, Colega MT, Na’im M, Ayob M, Robinson SM, Godfrey KM, et al. Identification and reproducibility of dietary patterns assessed with a FFQ among women planning pregnancy. Public Health Nutr. 2021;24(9):2437–46. 33745499 10.1017/S1368980021001178 PMC10195484

[12] Ta LDH, Chan JCY, Yap GC, Purbojati RW, Drautz-Moses DI, Koh YM, et al. A compromised developmental trajectory of the infant gut microbiome and metabolome in atopic eczema. Gut Microbes. 2020;12(1):1801964. 33023370 10.1080/19490976.2020.1801964 PMC7553750

[13] Martin M. Cutadapt removes adapter sequences from high-throughput sequencing reads. EMBnet J. 2011;17(1):10–2.

[14] Langmead B, Salzberg SL. Fast gapped-read alignment with Bowtie 2. Nat Methods. 2012;9(4):357–9. 22388286 10.1038/nmeth.1923 PMC3322381

[15] Buchfink B, Xie C, Huson DH. Fast and sensitive protein alignment using DIAMOND. Nat Methods. 2015;12(1):59–60. 25402007 10.1038/nmeth.3176

[16] Huson DH, Mitra S, Ruscheweyh H-J, Weber N, Schuster SC. Integrative analysis of environmental sequences using MEGAN4. Genome Res. 2011;21(9):1552–60. 21690186 10.1101/gr.120618.111 PMC3166839

[17] Beghini F, McIver LJ, Blanco-Míguez A, Dubois L, Asnicar F, Maharjan S, et al. Integrating taxonomic, functional, and strain-level profiling of diverse microbial communities with bioBakery 3. Elife. 2021;10:e65088. 33944776 10.7554/eLife.65088 PMC8096432

[18] Ng DZW, Yap GC, Tay CJX, Huang CH, Zhao S, Low A, et al. SPRESTO_Stool-AD. Github; 2026. https://github.com/davidhuy1408/SPRESTO_Stool-AD#.

[19] Cui H, Mu Z. Prenatal maternal risk factors contributing to atopic dermatitis: a systematic review and meta-analysis of cohort studies. Ann Dermatol. 2023;35(1):11. 36750454 10.5021/ad.21.268 PMC9905861

[20] Gough H, Grabenhenrich L, Reich A, Eckers N, Nitsche O, Schramm D, et al. Allergic multimorbidity of asthma, rhinitis and eczema over 20 years in the German birth cohort MAS. Pediatr Allergy Immunol. 2015;26(5):431–7. 26011739 10.1111/pai.12410 PMC4744942

[21] Miyoshi J, Hisamatsu T. The impact of maternal exposure to antibiotics on the development of child gut microbiome. Immunol Med. 2022;45(2):63–8. 34392799 10.1080/25785826.2021.1963189

[22] Azad MB, Konya T, Persaud RR, Guttman DS, Chari RS, Field CJ, et al. Impact of maternal intrapartum antibiotics, method of birth and breastfeeding on gut microbiota during the first year of life: a prospective cohort study. BJOG. 2016;123(6):983–93. 26412384 10.1111/1471-0528.13601

[23] Reduta T, Laudańska H, Laudanski P. Tissue inhibitors of matrix metalloproteinase‐1 levels are increased in serum of patients with allergic contact dermatitis. Contact Dermatitis. 2007;57(2):100–4. 17627649 10.1111/j.1600-0536.2007.01167.x

[24] Soyka MB, Wawrzyniak P, Eiwegger T, Holzmann D, Treis A, Wanke K, et al. Defective epithelial barrier in chronic rhinosinusitis: the regulation of tight junctions by IFN-γ and IL-4. J Allergy Clin Immunol. 2012;130(5):1087-96. e10. 22840853 10.1016/j.jaci.2012.05.052

[25] Chiricozzi A, Maurelli M, Peris K, Girolomoni G. Targeting IL-4 for the treatment of atopic dermatitis. Immunotargets Ther. 2020. 10.2147/ITT.S260370. 33062619 10.2147/ITT.S260370 PMC7532907

[26] Caspi R, Billington R, Fulcher CA, Keseler IM, Kothari A, Krummenacker M, et al. The MetaCyc database of metabolic pathways and enzymes. Nucleic Acids Res. 2018;46(D1):D633–9. 29059334 10.1093/nar/gkx935 PMC5753197

[27] Poston L, Godfrey KM, Gluckman PD, Hanson MA. Developmental origins of health and disease. Cambridge University Press; 2022. 10.1017/9781009272254.004.

[28] Vuillermin PJ, Macia L, Nanan R, Tang ML, Collier F, Brix S, editors. The maternal microbiome during pregnancy and allergic disease in the offspring. In: Seminars in immunopathology. Springer; 2017. 10.1007/s00281-017-0652-y. 10.1007/s00281-017-0652-y PMC5711986 29038841

[29] Du B, Shama A, Zhang Y, Chen B, Bu Y, Chen PA, et al. Gut microbiota and plasma metabolites in pregnant mothers and infant atopic dermatitis: a multi-omics study. World Allergy Organ J. 2025;18(1):101017. 39850616 10.1016/j.waojou.2024.101017 PMC11754505

[30] Ticer T, Stine R, Ellis T, Horvath TD, Haidacher SJ, Hoch KM, et al. Klebsiella pneumoniae cross‐feeds clostridioides difficile and enhances colonic pro‐inflammatory responses. FASEB J. 2022;36. 10.1096/fasebj.2022.36.S1.R5979.

[31] Molaaghaee-Rouzbahani S, Asri N, Sapone A, Baghaei K, Yadegar A, Amani D, et al. *Akkermansia**muciniphila* exerts immunomodulatory and anti-inflammatory effects on gliadin-stimulated THP-1 derived macrophages. Sci Rep. 2023;13(1):3237. 36828897 10.1038/s41598-023-30266-y PMC9958093

[32] Ottman N, Reunanen J, Meijerink M, Pietilä TE, Kainulainen V, Klievink J, et al. Pili-like proteins of Akkermansia muciniphila modulate host immune responses and gut barrier function. PLoS One. 2017;12(3):e0173004. 28249045 10.1371/journal.pone.0173004 PMC5332112

[33] Zhenyukh O, Civantos E, Ruiz-Ortega M, Sánchez MS, Vázquez C, Peiró C, et al. High concentration of branched-chain amino acids promotes oxidative stress, inflammation and migration of human peripheral blood mononuclear cells via mTORC1 activation. Free Radic Biol Med. 2017;104:165–77. 28089725 10.1016/j.freeradbiomed.2017.01.009

[34] Bredahl EC, Eckerson JM, Tracy SM, McDonald TL, Drescher KM. The role of creatine in the development and activation of immune responses. Nutrients. 2021;13(3):751. 33652752 10.3390/nu13030751 PMC7996722

[35] Munder M, Eichmann K, Morán JM, Centeno F, Soler G, Modolell M. Th1/Th2-regulated expression of arginase isoforms in murine macrophages and dendritic cells. J Immunol. 1999;163(7):3771–7. 10490974

[36] Levy BD, Noel PJ, Freemer MM, Cloutier MM, Georas SN, Jarjour NN, et al. Future research directions in asthma. An NHLBI working group report. Am J Respir Crit Care Med. 2015;192(11):1366–72. 26305520 10.1164/rccm.201505-0963WS PMC4731702

[37] Lebold KM, Drake MG, Hales-Beck LB, Fryer AD, Jacoby DB. IL-5 exposure in utero increases lung nerve density and airway reactivity in adult offspring. Am J Respir Cell Mol Biol. 2020;62(4):493–502. 31821769 10.1165/rcmb.2019-0214OC PMC7110978

[38] Chu HH, Kobayashi Y, Bui DV, Yun Y, Nguyen LM, Mitani A, et al. CCL4 regulates eosinophil activation in eosinophilic airway inflammation. Int J Mol Sci. 2022;23(24):16149. 36555793 10.3390/ijms232416149 PMC9782438

[39] Killian KN, Kosanovich JL, Lipp MA, Empey KM, Oury TD, Perkins TN. RAGE contributes to allergen driven severe neutrophilic airway inflammation via NLRP3 inflammasome activation in mice. Front Immunol. 2023;14:1039997. 36776857 10.3389/fimmu.2023.1039997 PMC9910358

[40] Manti S, Brown P, Perez M, Piedimonte G. The role of neurotrophins in inflammation and allergy. Vitam Horm. 2017;104:313–41. 28215300 10.1016/bs.vh.2016.10.010

[41] Nassenstein C, Braun A, Erpenbeck VJ, Lommatzsch M, Schmidt S, Krug N, et al. The neurotrophins nerve growth factor, brain-derived neurotrophic factor, neurotrophin-3, and neurotrophin-4 are survival and activation factors for eosinophils in patients with allergic bronchial asthma. J Exp Med. 2003;198(3):455–67. 12900521 10.1084/jem.20010897 PMC2194097

[42] Tokunaga R, Zhang W, Naseem M, Puccini A, Berger MD, Soni S, et al. CXCL9, CXCL10, CXCL11/CXCR3 axis for immune activation–a target for novel cancer therapy. Cancer Treat Rev. 2018;63:40–7. 29207310 10.1016/j.ctrv.2017.11.007 PMC5801162

[43] Huoman J, Haider S, Simpson A, Murray CS, Custovic A, Jenmalm MC. Childhood CCL18, CXCL10 and CXCL11 levels differentially relate to and predict allergy development. Pediatr Allergy Immunol. 2021;32(8):1824–32. 34101271 10.1111/pai.13574 PMC11497305

[44] Guo L, Li N, Yang Z, Li H, Zheng H, Yang J, et al. Role of CXCL5 in regulating chemotaxis of innate and adaptive leukocytes in infected lungs upon pulmonary influenza infection. Front Immunol. 2021;12:785457. 34868067 10.3389/fimmu.2021.785457 PMC8637413

[45] Rothers J, Stern DA, Lohman IC, Spangenberg A, Wright AL, DeVries A, et al. Maternal cytokine profiles during pregnancy predict asthma in children of mothers without asthma. Am J Respir Cell Mol Biol. 2018;59(5):592–600. 29863910 10.1165/rcmb.2017-0410OC PMC6236694

[46] Robert-McComb JJ, González ÁG, Carraway L. Nutritional guidelines and energy needs during pregnancy and lactation. In: The active female: health issues throughout the lifespan. 2014. p. 517–33.

[47] Abrahamsson TR, Jakobsson HE, Andersson AF, Björkstén B, Engstrand L, Jenmalm MC. Low diversity of the gut microbiota in infants with atopic eczema. J Allergy Clin Immunol. 2012;129(2):434-402. e2. 22153774 10.1016/j.jaci.2011.10.025

[48] Stewart CJ, Ajami NJ, O’Brien JL, Hutchinson DS, Smith DP, Wong MC, et al. Temporal development of the gut microbiome in early childhood from the TEDDY study. Nature. 2018;562(7728):583–8. 30356187 10.1038/s41586-018-0617-x PMC6415775

[49] Hickman B, Salonen A, Ponsero AJ, Jokela R, Kolho KL, de Vos WM, et al. Gut microbiota wellbeing index predicts overall health in a cohort of 1000 infants. Nat Commun. 2024;15(1):8323. 39333099 10.1038/s41467-024-52561-6 PMC11436675

[50] Lay C, Chu CW, Purbojati RW, Acerbi E, Drautz-Moses DI, de Sessions PF, et al. A synbiotic intervention modulates meta-omics signatures of gut redox potential and acidity in elective caesarean born infants. BMC Microbiol. 2021;21(1):191. 34172012 10.1186/s12866-021-02230-1 PMC8229302

[51] Barcenilla A, Pryde SE, Martin JC, Duncan SH, Stewart CS, Henderson C, et al. Phylogenetic relationships of butyrate-producing bacteria from the human gut. Appl Environ Microbiol. 2000;66(4):1654–61. 10742256 10.1128/aem.66.4.1654-1661.2000 PMC92037

[52] Zhang D, Jian YP, Zhang YN, Li Y, Gu LT, Sun HH, et al. Short-chain fatty acids in diseases. Cell Commun Signal. 2023;21(1):212. 37596634 10.1186/s12964-023-01219-9 PMC10436623

[53] Ezzine C, Loison L, Montbrion N, Bôle-Feysot C, Déchelotte P, Coëffier M, et al. Fatty acids produced by the gut microbiota dampen host inflammatory responses by modulating intestinal SUMOylation. Gut Microbes. 2022;14(1):2108280. 35978476 10.1080/19490976.2022.2108280 PMC9466625

[54] Fujimura T, Lum SZC, Nagata Y, Kawamoto S, Oyoshi MK. Influences of maternal factors over offspring allergies and the application for food allergy. Front Immunol. 2019;10:466809. 10.3389/fimmu.2019.01933 PMC6716146 31507589

[55] Schäfer S, Liu A, Campbell D, Nanan R. Analysis of maternal and perinatal determinants of allergic sensitization in childhood. Allergy Asthma Clin Immunol. 2020;16(1):71. 32922456 10.1186/s13223-020-00467-5 PMC7477859

[56] Lu X, Shi Z, Jiang L, Zhang S. Maternal gut microbiota in the health of mothers and offspring: from the perspective of immunology. Front Immunol. 2024;15:1362784. 38545107 10.3389/fimmu.2024.1362784 PMC10965710

[57] Rudi K, Storrø O, Øien T, Johnsen R. Modelling bacterial transmission in human allergen‐specific IgE sensitization. Lett Appl Microbiol. 2012;54(5):447–54. 22385401 10.1111/j.1472-765X.2012.03229.x

[58] Funkhouser LJ, Bordenstein SR. Mom knows best: the universality of maternal microbial transmission. PLoS Biol. 2013;11(8):e1001631. 23976878 10.1371/journal.pbio.1001631 PMC3747981

[59] Zainal NHM, Nor NHM, Saat A, Clifton VL. Childhood allergy susceptibility: the role of the immune system development in the in-utero period. Hum Immunol. 2022;83(5):437–46. 35183391 10.1016/j.humimm.2022.02.002

[60] Zhang H, Zhang Z, Liao Y, Zhang W, Tang D. The complex link and disease between the gut microbiome and the immune system in infants. Front Cell Infect Microbiol. 2022;12:924119. 35782111 10.3389/fcimb.2022.924119 PMC9241338

[61] Garrett WS, Gordon JI, Glimcher LH. Homeostasis and inflammation in the intestine. Cell. 2010;140(6):859–70. 20303876 10.1016/j.cell.2010.01.023 PMC2845719

[62] El‐Heis S, D’Angelo S, Curtis EM, Healy E, Moon RJ, Crozier SR, et al. Maternal antenatal vitamin D supplementation and offspring risk of atopic eczema in the first 4 years of life: evidence from a randomized controlled trial. Br J Dermatol. 2022;187(5):659–66. 35763390 10.1111/bjd.21721 PMC9804289

[63] Ng DZW, Yap GC, Tay CJX, Huang C-H, Zhao S, Low A, et al. Maternal-prenatal signature underlying offspring atopic dermatitis susceptibility. BioProject. 2025. https://www.ncbi.nlm.nih.gov/bioproject/PRJNA1273620/.

