# Supplementary material for: Maternal-prenatal gut microbiome-systemic metabolome perturbations and TH2-skewed immunity link to offspring gut microbiome disruption and atopic dermatitis susceptibility
Source: Genome Med. 2026 Apr 17;18:72. doi: 10.1186/s13073-026-01655-5 (PMC13214156; doi:10.1186/s13073-026-01655-5)
Supplement: Supplementary file 1 — Additional file 1: Table S1. Genomic information and phylogenetic identity of the Klebsiella pneumoniae strains. Table S2. Comparison of demographic and clinical characteristics between infants from control and AD groups during various timepoints during early-life. Table S3. Comparison of nutrient and food group data between mothers from control and AD groups at the third trimester of pregnancy. Table S4. Differential bacteria between mothers from control and AD groups which were identified at both first and third trimester of pregnancy. Table S5. Enriched or depleted functional gene carriage between mothers from control and AD groups at both first and third trimesters of pregnancy. Table S6. Targeted plasma metabolomics of specific metabolites identified from functional genomic pathways of differential gut bacteria between groups, were performed in mothers at third trimester (27-28 weeks) of pregnancy. Table S7. Comparison of maternal-prenatal immune markers between groups across five pregnancy timepoints. Table S8. Differential gut bacteria and perturbed stool metabolites between control and AD infants during offspring early life. Figure S1. KEGG biosynthesis pathways for valine, leucine and isoleucine. Figure S2. Community-level gut microbiome diversity across prenatal and postnatal timepoints. Figure S3. In vitro mechanistic experiments demonstrating (A) bacterial mediated synthesis of gut microbial-derived metabolites and (B) flux of these across an in vitro cellular colonic model. [file 13073_2026_1655_MOESM1_ESM.docx]

**SUPPLEMENTARY METHODS**

*Bacterial culture growth conditions and BCAA biosynthesis experiment*

*Roseburia intestinalis* DSM 14610^T^ was acquired from DSMZ, the German culture collection. The three *Klebsiella pneumoniae* strains WHN643, WHN880 and WHN1212 originated from terminal ileal washings of three healthy patients who underwent colonoscopy at the National University Hospital, Singapore with patients’ informed consents and ethics approvals from the Domain Specific Review Board of the National Healthcare Group (ID:2015/00780).

*R. intestinalis* was handled and incubated statically at 37^o^C in a Coy anaerobic chamber. *R. intestinalis* was incubated for 20h in 20 mL YCFA broth where OD600nm=2.33 ± 0.01 technical triplicates was used for the BCAA experiment [1]. *Klebsiella pneumoniae* strains were streaked from glycerol stocks onto MacConkey agar (Sigma, product no. M7408) and cultured in Lysogeny broth (LB) [2]. For the BCAA biosynthesis experiment, the *Klebsiella* strains were cultivated in 20 mL LB overnight at 37^o^C with shaking at 150 rpm and harvested when the mean OD600nm was 3.6 ± 0.05 (n=3).

The BCAA biosynthesis experiment was performed according to condition 4 as previously described with a modified assay buffer [3]. All cultures were washed twice with L-cysteine (0.1% w/v) reduced Phosphate buffer saline (PBS; Viantis, product number PB0344) pH 7.2 buffer to remove residual BCAA prior to the experiment. Briefly, cell cultures were centrifuged at 5,000 × g for 10 min, and the supernatant was discarded. The cell pellets were resuspended in an equal volume of PBS, followed by a second centrifugation under the same conditions. After removing the supernatant, the washed cells were resuspended in an equal volume of assay buffer (2 ml). The assay buffer was prepared using reduced PBS that contained 5 g/L (NH_4_)_2_SO_4_ (Sigma, product no. A4915), 0.1 g/L CaCl_2_ · 2H_2_O (Sigma, product no. 223506), 0.1 g/L MgCl2 · 6H_2_O (Sigma, product no. [**M2670**](https://www.sigmaaldrich.com/SG/en/product/sial/m2670)), 5 g/L D-(+)-Glucose (Sigma, product no. G5767), 1 × trace mineral supplement (ATCC, product ID MD-TMS), 1 × vitamin supplement (ATCC, product ID MD-VS), and 1 g/L vitamin K_3_ (Sigma, product no. M5625). 200 µl samples were collected at 0, 30, and 60 mins. Each sample was immediately mixed with an equal volume of acetonitrile (1:1, v/v) to quench the reaction, followed by centrifugation at 10, 000 rpm for 5 mins to isolate the supernatant for subsequent LC-MS/MS analysis. L-tryptophan (indole-D5, 98%) (Cambridge Isotope Laboratories, Inc., product nu. DLM-1092) was used as internal standard (IS). All experiments were conducted in biological replicates under anaerobic conditions within an anaerobic chamber.

*Genome sequencing, assembly and annotation*

Total DNA for shotgun sequencing was extracted from 4 mL of *K. pneumoniae* cultures (OD600nm ~3.6). The PowerFecal Pro DNA kit (Qiagen, cat. no. 51804) was used to extract genomic DNA as previously described [4]. Shotgun library prep and sequencing was performed by an external vendor (AvernirBio) using the VAHTS Universal DNA Library Prep Kit for Illumina V4 v22.1 (Vazyme) for 150bp paired-end sequencing and NovaSeqX Plus 25B flow cell (Illumina), respectively. The raw reads of each genome were quality checked using fastp v0.23.2 as previously described [4, 5]. Filtered reads were used for genome assembly using SPAdes v3.15.5 [6]. The draft genomes were further screened for contaminating adapters and sequences using NCBI Foreign Contamination Screen-adapter and GX v3.4.0 on the Galaxy platform v0.5.0 [7, 8]. Prokka v1.14.5 was used to annotate proteins, genes and rRNA from the draft genomes [9]. After which, CheckM v1.1.3 was used to assess percentage completeness and contamination [10]. *K. pneumoniae* sequence typing was performed using MLST v2.0.9 database updated 2023-June-19 [11]. Taxonomic identity was verified based on highest digital DNA-DNA hybridization (dDDH) value calculated using the d4 formula and <1% G+C content difference against genomes of type strains in the Type(Strain) Genome Server (accessed 2024-Dec-26) [12, 13]. **Supplementary table 1** shows the sequencing coverage, genomic, sequence typing and taxonomic information for the *K. pneumoniae* strains.

*KEGG annotation of BCAA biosynthesis pathway*

*R. intestinalis* DSM 14610^T^ genome (RefSeq no. GCF_000156535.1) predicted protein sequences were downloaded from the NCBI dataset [14]. The BCAA biosynthesis pathway for all strains was identified using BLASTKoala v3.1 against the KEGG database updated 2024-Dec-01 accessed 2024-Dec-27 [15].

*Caco-2 cell culture.*

Caco-2 cells were obtained from American Type Culture Collection (ATCC® HTB-37). Caco-2 cells were cultured in Minimum Essential Media (MEM) complete media containing 1% L-glutamine (PAN-Biotech (Bavaria, Germany)), 1% non-essential amino acids (MP Biomedicals ( Irvine, CA, USA)), 1% sodium pyruvate (ThermoFisher Scientific (Waltham, MA, USA)) and 2% sodium bicarbonate (HiMedia Laboratories (Mumbai, India)), and supplemented with 20% faetal bovine serum (FBS; HyClone Laboratories (Logan, UT, USA)) and 1% penicillin-streptomycin (HyClone Laboratories (Logan, UT, USA)). Cell cultures were maintained in an incubator under standard conditions (37°C, humidified atmosphere with 5% CO_2_).

*Caco-2 transwell assay.*

Transwell assays were performed following previously validated experimental protocols [16] using six biological replicates. Prior to each experiment, Caco-2 cells (passage number 30-40) were seeded on culture plate well inserts (Greiner Bio-one; Product no. 662 610) at a cell density of 100,000 cells/insert and grown for 21 days to achieve confluent and highly differentiated cell monolayers. Prior to each experiment, culture media was removed from both chambers and each insert was washed with 500 µL phosphate buffer solution (PBS; HyClone Laboratories (Logan, UT, USA)) before being transferred to a new 24 well plate containing transport buffer.

*LC-MS/MS analyses of BCAA, atenolol and propranolol*

LC-MS/MS analyses was performed using an Agilent 1290 Infinity liquid chromatography system (Agilent Technologies, Santa Clara, CA, USA) coupled to an AB SCIEX 5500 QTRAP hybrid linear ion-trap quadrupole mass spectrometer equipped with a Turbo Ion Spray source (Applied Biosystems, Foster City, CA, USA). Analyst software, version 1.7.1, according to a modified protocol adapted from [17, 18] under positive mode electrospray ionization (ESI) with multiple reaction monitoring (MRM) experiment.

Atlantis Premier BEH C18 AX 2.1 × 100 mm, 1.7 μm column (Waters Corp., Milford, MA, USA) was utilized as the stationary phase, while mobile phases A and B were water with 0.1% formic acid and acetonitrile with 0.1% formic acid respectively. The elution gradient was set as: linear gradient 5-65% B (0.00-2.50 min), linear gradient 65-5% B (2.50-2.51 min), isocratic 5% B (2.51-3.50 min) at a constant flow rate of 0.4 mL/min. The autosampler was thermostatted at 4°C and 2 μL of sample was injected into the column for each sample. The temperature of the column was maintained at 55°C and the autosampler needle was flushed with 100% water after every injection to prevent carryover.

MS source parameters were programmed as such: ion spray voltage= 4500 V; source temperature = 500°C; collision gas (N_2_) setting = medium; ion source gas 1 (N_2_) = 20 psi; ion source gas 2 (N_2_) = 20 psi; curtain gas (N_2_) = 20 psi; dwell time = 100 ms. MultiQuant software version 3.0.3 (Applied Biosystems, Foster City, CA, USA) was used to quantitate all acquired sample chromatograms. Analytes were quantitated as their peak area ratio based on the ratio of peak area of each analyte to peak area of IS (Tryptophan (indole-D5, 98%)).

**SUPPLEMENTARY DATA**

**Table S1:** Genomic information and phylogenetic identity of the *Klebsiella pneumoniae* strains.

| **Strain name** | **WHN643** | **WHN880** | **WHN1212** |
| --- | --- | --- | --- |
| BioProject/Biosample/Genome accession numbers | PRJNA1157478/SAMN45967172/ JBKGTU000000000 | PRJNA1157478/SAMN45967173/ JBKFTC000000000 | PRJNA1157478/SAMN45967174/ JBKFTD000000000 |
| Genome size (bp) | 5,656,139 | 5,705,877 | 5,989,007 |
| Total raw paired-end reads | 73,945,556 | 53,979,102 | 55,649,070 |
| Coverage × (filtered read count × read length / total genome size) | 1,961 | 1,419 | 1,394 |
| No. of N base | 1,200 | 1,100 | 1,000 |
| No. of scaffolds ≥200 bp | 1,145 | 591 | 2,286 |
| No. of genes | 5,124 | 5,301 | 5,317 |
| No. of CDS | 5,035 | 5,225 | 5,223 |
| rRNAs (5S, 16S, 23S) | 8 (3, 3 partial, 2 partial) | 6 (2, 2 partial, 2 partial) | 6 (2, 2 partial, 2 partial) |
| tRNAs | 80 | 78 | 87 |
| tmRNA | 1 | 1 | 1 |
| G+C content (%) | 57.1 | 57.0 | 56.2 |
| N50 assembled genome (bp) | 317,779 | 366,115 | 257,389 |
| CheckM Completeness (%) | 99.7 | 100 | 100 |
| CheckM Contamination (%) | 3.82 | 1.5 | 4.82 |
| dDDH to specified taxon (<1% G+C content difference) | 93.0% to *Klebsiella pneumoniae* ATCC 13883^T^ (0.05%) | 92.8% to *Klebsiella pneumoniae*  subsp. *ozaenae* ATCC  11296^T^ (0.25%) | 92.8% to *Klebsiella pneumoniae* ATCC 13883^T^ (0.84%) |
| Sequence Type | 86 | 11 | 1401 |

**Table S2:** Comparison of demographic and clinical characteristics between infants from control and AD groups during various timepoints during early-life.

| **Demographics and clinical characteristics** | **Week 3** | | | | **Month 3** | | | | **Month 6** | | | |
| --- | --- | --- | --- | --- | --- | --- | --- | --- | --- | --- | --- | --- |
|  | **Control (n=20)** | | **AD (n=11)** | | **Control (n=16)** | | **AD (n=13)** | | **Control (n=30)** | | **AD (n=25)** | |
|  | n | % | n | % | n | % | n | % | n | % | n | % |
| Antibiotics during pregnancy | 6 | 35.3 | 3 | 27.3 | 3 | 18.8 | 5 | 38.5 | 3 | 10.0 | 9 | 36.0 |
| Preterm birth | 0 | 0.00 | 0 | 0.00 | 2 | 12.5 | 0 | 0.00 | 1 | 3.33 | 0 | 0.00 |
| Born by caesarean delivery | 3 | 15.0 | 5 | 45.5 | 3 | 18.8 | 6 | 46.2 | 3 | 10.0 | 10 | 40.0 |
| Sex (male) | 12 | 60.0 | 5 | 45.5 | 9 | 56.3 | 9 | 69.2 | 18 | 60 | 15 | 60.0 |
| Presence of siblings | 8 | 47.1 | 5 | 55.6 | 4 | 25.0 | 5 | 38.5 | 8 | 26.7 | 8 | 32.0 |
| Undergone phototherapy | 10 | 66.7 | 6 | 75.0 | 7 | 43.8 | 3 | 23.1 | 12 | 40.0 | 12 | 48.0 |
| Owns a pet | 2 | 11.8 | 1 | 9.09 | 2 | 12.5 | 5 | 38.5 | 4 | 13.3 | 5 | 20.0 |
| Antibiotic intake | - | - | - | - | 1 | 6.25 | 1 | 7.69 | - | - | - | - |
| **Maternal clinical history** |  |  |  |  |  |  |  |  |  |  |  |  |
| History of allergy | 11 | 55.0 | 8 | 72.7 | **6*** | **37.5*** | **11*** | **84.6*** | 17 | 56.7 | 19 | 76.0 |
| **Ethnicity** |  |  |  |  |  |  |  |  |  |  |  |  |
| Chinese | 15 | 75.0 | 10 | 90.9 | 12 | 75.0 | 11 | 84.6 | 24 | 80.0 | 21 | 84.0 |
| Malay | 4 | 20.0 | 1 | 9.09 | 3 | 18.8 | 2 | 15.4 | 3 | 10.0 | 4 | 16.0 |
| Indian | 0 | 0.00 | 0 | 0.00 | 0 | 0.00 | 0 | 0.00 | 1 | 3.33 | 0 | 0.00 |
| Mixed | 1 | 5.00 | 0 | 0.00 | 1 | 6.25 | 0 | 0.00 | 2 | 6.67 | 0 | 0.00 |
| **Feeding history** |  |  |  |  |  |  |  |  |  |  |  |  |
| Exclusive breastfeeding | 6 | 30.0 | 2 | 18.2 | 6 | 37.5 | 3 | 23.1 | 10 | 33.3 | 12 | 48.0 |
| Breastfeeding and formula | 14 | 70.0 | 7 | 63.6 | 10 | 62.5 | 9 | 69.2 | 19 | 63.3 | 12 | 48.0 |
| Exclusive formula | 0 | 0.00 | 2 | 18.2 | 0 | 0.00 | 0 | 0.00 | 0 | 0.00 | 0 | 0.00 |

Data is provided in n (%) for categorical data. Statistical analyses were performed between groups at specific timepoints using Chi squared test for categorical variables. **Bolded values with *** represent statistically significant differences between groups (*p*<0.05).

**Table S3:** Comparison of nutrient and food group data between mothers from control and AD groups at the third trimester of pregnancy.

| **Maternal dietary intake at third trimester of pregnancy** | | **Control** | **AD** | ***p*** |
| --- | --- | --- | --- | --- |
| **Nutrient intake (normalized by 1000kcal)** | Carbohydrate (g) | 132 ± 13.6 | 131 ± 11.4 | ns |
|  | Protein (g) | 38.7 ± 5.86 | 39.5 ± 6.08 | ns |
|  | Fat (g) | 33.9 ± 5.49 | 34.0 ± 4.74 | ns |
|  | Cholesterol (mg) | 108 ± 38.2 | 119 ± 78.8 | ns |
|  | Fibre (g) | 11.6 ± 2.27 | 11.7 ± 1.87 | ns |
|  | Saturated fats (g) | 15.3 ± 2.95 | 16.0 ± 2.19 | ns |
|  | Monounsaturated fats (g) | 11.0 ± 2.36 | 10.7 ± 2.29 | ns |
|  | Polyunsaturated fats (g) | 5.01 ± 1.26 | 4.69 ± 1.12 | ns |
|  | Sodium (mg) | 1260 ± 297 | 1290 ± 287 | ns |
|  | Calcium (mg) | 651 ± 372 | 772 ± 447 | ns |
|  | Iron (mg) | 11.0 ± 5.85 | 12.3 ± 7.33 | ns |
|  | Vitamin A (µg) | 599 ± 260 | 680 ± 284 | ns |
|  | Beta-carotene (µg) | 2425 ± 1923 | 2526 ± 1777 | ns |
| **Food group data^1^ (normalized by 1000kcal)** | White bread | 3.22 ± 8.89 | 3.40 ± 6.19 | ns |
|  | Wholemeal bread | 8.00 ± 9.23 | 3.59 ± 7.66 | ns |
|  | Bread spreads | 0.97 ± 2.27 | 0.73 ± 2.03 | ns |
|  | Ethnic breads and buns | 6.65 ± 14.7 | 7.84 ± 11.8 | ns |
|  | White rice | 53.1 ± 49.2 | 42.1 ± 40.1 | ns |
|  | Flavoured rice | 8.71 ± 11.9 | 8.18 ± 11.7 | ns |
|  | Vegetables (Less healthy) | 0.00 ± 11.0 | 0.00 ± 3.97 | ns |
|  | Meat (Less healthy) | 2.79 ± 3.83 | 2.23 ± 3.15 | ns |
|  | Desserts (Sweet snacks) | 30.5 ± 21.3 | 21.0 ± 23.3 | ns |
|  | Fast food (Fried salty food) | 25.0 ± 21.0 | 17.9 ± 14.7 | ns |
|  | Dairy without milk | 33.3 ± 51.3 | 39.7 ± 34.5 | ns |
|  | Sweetened beverages | 27.7 ± 36.2 | 31.4 ± 42.6 | ns |
|  | Decaffeinated coffee or tea | 30.1 ± 71.3 | 16.1 ± 62.3 | ns |
|  | Cereals, oats and cereal drinks) | 3.72 ± 32.9 | 3.07 ± 21.8 | ns |
|  | Full fat milk | 0.00 ± 60.3 | 3.92 ± 93.8 | ns |
|  | Formula (Low fat milk) | 34.0 ± 48.3 | 40.5 ± 65.0 | ns |
|  | Brown rice | 4.65 ± 30.0 | 0.00 ± 28.9 | ns |
|  | Soups | 23.8 ± 27.0 | 29.1 ± 29.1 | ns |
|  | Vegetables (Healthy) | 69.9 ± 52.1 | 75.2 ± 44.8 | ns |
|  | All fruits | 45.9 ± 47.3 | 51.7 ± 42.2 | ns |
|  | Meat (Healthy) | 10.3 ± 7.32 | 9.53 ± 8.34 | ns |
|  | Fish and seafood | 15.7 ± 22.1 | 14.4 ± 13.5 | ns |
|  | Eggs | 11.3 ± 10.0 | 11.0 ± 19.4 | ns |
|  | Plant protein | 19.2 ± 21.9 | 22.5 ± 43.0 | ns |
|  | Porridge | 9.56 ± 39.4 | 6.50 ± 41.8 | ns |
|  | Noodles and pasta | 81.9 ± 63.0 | 85.8 ± 73.0 | ns |
|  | Soft drinks | 0.00 ± 17.0 | 0.00 ± 14.5 | ns |

Data shown are presented as geometric mean **±** standard deviation (SD). Statistical analyses were conducted between groups at specific time points using Mann-Whitney U test. ^1^Units for intake of specific food groups are expressed in grams (g).

**Table S4:** Differential bacteria between mothers from control and AD groups which were identified at both first and third trimester of pregnancy.

| **Species** | **First trimester** | | | **Third trimester** | | | **Longitudinal *p*** | **FDR-adjusted longitudinal p** |
| --- | --- | --- | --- | --- | --- | --- | --- | --- |
|  | **Control** | **AD** | ***p*** | **Control** | **AD** | ***p*** |  |  |
| *Klebsiella pneumoniae* | 10,863 ± 7,590 | 13,185 ± 13,131 | **0.019** | 10,722 ± 41,572 | 17,523 ± 10,335 | **0.016** | **0.017** | **0.028** |
| *Romboutsia timonensis* | 17,678 ± 11,529 | 15,371 ± 14,623 | **0.023** | 21,955 ± 15,159 | 13,888 ± 13,057 | **0.022** | **0.013** | **0.025** |
| *Akkermansia muciniphila* | 20,591 ± 15,864 | 10,543 ± 7,285 | **0.029** | 12,788 ± 12,881 | 6,155 ± 5,490 | ns | **0.012** | **0.025** |
| *Roseburia intestinalis* | 7,321 ± 6,963 | 17,278 ± 13,707 | ns | 8,680 ± 7,742 | 17,210 ± 12,747 | **0.019** | **0.012** | **0.025** |
| *Clostridioides difficile* | 5,997 ± 2,160 | 6,943 ± 2,401 | **0.033** | 5,965 ± 2,281 | 6,900 ± 2,728 | **0.040** | **0.011** | **0.023** |
| *Clostridia bacterium* | 4,861 ± 4,058 | 6,788 ± 5,604 | ns | 6,576 ± 6,825 | 6,123 ± 6,769 | **0.048** | ns | ns |
| *Tyzzerella nexilis* | 3,270 ± 3,345 | 3,163 ± 2,981 | ns | 2,511 ± 2,588 | 4,652 ± 4,386 | **0.010** | ns | ns |
| *Bilophila* sp. 4_1_30 | 3,743 ± 3,161 | 4,042 ± 3,790 | **0.041** | 4,199 ± 4,999 | 4,850 ± 4,387 | ns | **0.035** | ns |
| *Blautia hansenii* | 4,625 ± 3,497 | 3,060 ± 3,125 | **0.018** | 3,871 ± 3,350 | 2,794 ± 2,137 | **0.046** | **0.017** | **0.028** |
| *Alistipes ihumii* | 5,658 ± 5,653 | 3,046 ± 3,757 | **0.034** | 4,110 ± 4,369 | 1,896 ± 1,300 | **0.021** | **0.034** | **0.047** |
| *Enterococcus avium* | 760 ± 885 | 80 ± 193 | ns | 17 ± 64 | 326 ± 225 | **0.038** | ns | ns |
| *Firmicutes bacterium* CAG:94 | 3,615 ± 3,640 | 4,770 ± 3,568 | ns | 4,372 ± 4,050 | 1,938 ± 1,991 | **0.009** | ns | ns |
| *Veillonella parvula* | 115 ± 273 | 144 ± 305 | ns | 99 ± 235 | 237 ± 267 | **0.039** | ns | ns |
| *Clostridium* sp. CAG:678 | 3,154 ± 3,153 | 1,679 ± 1,459 | **0.039** | 4,552 ± 4,200 | 3,299 ± 3,645 | **0.012** | **0.013** | **0.025** |

Data shown are the normalized reads of bacteria and are presented as geometric mean **±** standard deviation (SD). Statistical analyses were conducted between groups at specific time points using the Mann-Whitney U test. Longitudinal analyses across time points were performed using a linear mixed-effects model adjusting for maternal history of allergy (prenatal period), with multiple testing controlled using a false discovery rate (FDR) of 10%.

**Table S5:** Enriched or depleted functional gene carriage between mothers from control and AD groups at both first and third trimesters of pregnancy.

| **Direction of perturbation** | **Functional gene carriage**  **(from Metacyc)** | **First trimester** | | | **Third trimester** | | | **Longitudinal *p*** | **FDR-adjusted longitudinal p** |
| --- | --- | --- | --- | --- | --- | --- | --- | --- | --- |
|  |  | **Control** | **AD** | ***p*** | **Control** | **AD** | ***p*** |  |  |
| Enriched in AD group | Glycolysis III (from glucose) | 2.62 e^-6^ ± 6.11 e^-6^ | 4.99 e^-6^ ± 1.01 e^-6^ | ns | 5.03 e^-6^ ± 1.33 e^-6^ | 5.11 e^-6^ ± 8.19 e^-6^ | ns | **0.032** | **0.048** |
|  | Creatinine degradation I | 6.10 e^-8^ ± 3.07 e^-8^ | 1.08 e^-7^ ± 6.26 e^-7^ | ns | 2.33 e^-8^ ± 2.44 e^-8^ | 3.29 e^-7^ ± 7.03 e^-7^ | **0.026** | **0.020** | **0.039** |
|  | Superpathway of BCAA biosynthesis | 3.76 e^-6^ ± 9.83 e^-6^ | 7.12 e^-6^ ± 6.19 e^-6^ | **0.030** | 3.82 e^-6^ ± 5.78 e^-6^ | 4.50 e^-6^ ± 4.85 e^-6^ | ns | **0.029** | **0.045** |
|  | L-Isoleucine biosynthesis I (from threonine) | 3.74 e^-5^ ± 1.00 e^-5^ | 7.14 e^-5^ ± 6.13 e^-5^ | **0.024** | 3.82 e^-5^ ± 5.86 e^-5^ | 4.53 e^-5^ ± 4.93 e^-5^ | ns | **0.028** | **0.045** |
|  | L-Valine biosynthesis | 6.57 e^-5^ ± 1.11 e^-5^ | 9.49 e^-5^ ± 8.95 e^-5^ | **0.028** | 5.15 e^-5^ ± 7.95 e^-5^ | 6.75 e^-5^ ± 5.78 e^-5^ | ns | **0.015** | **0.032** |
| Depleted in AD group | L-Arginine biosynthesis II (acetyl cycle) | 7.43 e^-5^ ± 1.01 e^-5^ | 3.83 e^-6^ ± 6.41 e^-6^ | **0.030** | 4.52 e^-5^ ± 5.93 e^-5^ | 3.95 e^-6^ ± 5.18 e^-6^ | ns | **0.035** | **0.048** |
|  | Aerobactin biosynthesis | 1.52 e^-7^ ± 9.87 e^-7^ | 3.20 e^-8^ ± 2.36 e^-8^ | ns | 6.49 e^-7^ ± 2.84 e^-7^ | 1.41 e^-8^ ± 5.95 e^-8^ | **0.017** | **0.021** | **0.039** |

Data shown are the normalized reads of functional genes and are presented as geometric mean **±** standard deviation (SD). Statistical analyses were conducted between groups at specific time points using the Mann-Whitney U test. Longitudinal analyses across time points were performed using a linear mixed-effects model adjusting for maternal history of allergy (prenatal period), with multiple testing controlled using a false discovery rate (FDR) of 10%.

**Table S6:** Targeted plasma metabolomics of specific metabolites identified from functional genomic pathways of differential gut bacteria between groups, were performed in mothers at third trimester (27-28 weeks) of pregnancy.

| **Functional gene carriage (from Metacyc)** | **Metabolites involved** | **Control** | **AD** | ***p*** | **FDR-adjusted p** |
| --- | --- | --- | --- | --- | --- |
| Glycolysis III | Pyruvate | 105 (93.8-128) | 125 (99.0-153) | **0.005** | **0.003** |
| Creatine degradation I | Creatinine | 47.1 (44.5-52.4) | 45.9 (43.8-49.2) | ns | ns |
|  | Creatine | 44.5 (36.3-53.3) | 49.2 (44.1-55.4) | **0.032** | **0.048** |
|  | Glycine | 124 (109-136) | 125 (114-141) | ns | ns |
| Superpathway of BCAA biosynthesis, L-Isoleucine biosynthesis I (from threonine), L-Valine biosynthesis | Threonine | 122 (104-146) | 117 (101-130) | ns | ns |
|  | Leucine | 79.3 (72.3-85.0) | 81.0 (74.9-91.4) | **0.034** | **0.048** |
|  | Isoleucine | 44.7 (40.9-48.7) | 47.5 (42.9-52.1) | **0.010** | **0.030** |
|  | Valine | 165 (149-179) | 177 (162-195) | **0.002** | **0.016** |
| L-Arginine biosynthesis II | Glutamate | 51.3 (42.2-62.8) | 57.7 (42.9-68.9) | ns | ns |
|  | Glutamine | 352 (329-387) | 358 (334-382) | ns | ns |
|  | Ornithine | 24.3 (21.8-27.8) | 27.1 (22.7-30.4) | **0.029** | **0.039** |
|  | Arginine | 35.5 (32.2-38.9) | 35.2 (30.4-38.6) | ns | ns |
| Aerobactin synthesis | Lysine | 142 (129-157) | 151 (143-159) | **0.019** | **0.029** |

Data shown are the concentrations of metabolites (µM) and are presented as median (interquartile range: 25^th^ – 75^th^ percentile). Between-group comparisons were performed using the Mann–Whitney U test, with multiple testing controlled using a false discovery rate (FDR) of 10%. Bolded values indicate statistically significant differences (FDR-adjusted p < 0.05).

**Table S7:** Comparison of maternal-prenatal immune markers between groups across five pregnancy timepoints.

|  | **6-8 weeks** | | | **11-13 weeks** | | | **18-21 weeks** | | | **27-28 weeks** | | | **34-36 weeks** | | | **Longitudinal analyses (mixed model linear regression)** | | |
| --- | --- | --- | --- | --- | --- | --- | --- | --- | --- | --- | --- | --- | --- | --- | --- | --- | --- | --- |
|  | **Control** | **AD** | ***p*** | **Control** | **AD** | ***p*** | **Control** | **AD** | ***p*** | **Control** | **AD** | ***p*** | **Control** | **AD** | ***p*** | **Longitudinal *p*** | **FDR-adjusted Longitudinal *p*** |  |
| IL8 | 6.11 (5.92-6.51) | 6.28 (5.75-6.84) | ns | 6.26 (5.76-6.56) | 6.26 (5.77-6.58) | ns | 5.98 (5.73-6.38) | 6.03 (5.69-6.48) | ns | 6.18 (5.89-6.53) | 6.15 (5.66-6.5) | ns | 6.53 (6.18-6.97) | 6.45 (6.16-6.97) | ns | ns | ns |  |
| VEGFA | 11.18 (11.01-11.49) | 11.18 (10.95-11.47) | ns | 11.28 (11.12-11.52) | 11.27 (11.02-11.51) | ns | 11.51 (11.34-11.64) | 11.44 (11.24-11.68) | ns | 11.59 (11.33-11.8) | 11.55 (11.33-11.79) | ns | 11.6 (11.35-11.92) | 11.63 (11.39-11.85) | ns | ns | ns |  |
| CD8A | 10.74 (10.15-11.16) | 10.97 (10.44-11.43) | **0.088** | 10.36 (9.97-10.89) | 10.72 (10.08-11.01) | **0.096** | 10.11 (9.51-10.56) | 10.14 (9.79-10.49) | ns | 9.64 (8.8-10.06) | 9.6 (9.26-10.12) | ns | 9.07 (8.38-9.69) | 9.26 (8.85-9.83) | ns | ns | ns |  |
| MCP-3 | 2.22 (1.84-2.46) | 2.39 (2.05-2.61) | **0.057** | 2.12 (1.85-2.4) | 2.21 (2.03-2.64) | ns | 2.05 (1.77-2.35) | 2.16 (1.76-2.59) | ns | 2.12 (1.77-2.5) | 2.21 (1.82-2.61) | ns | 2.16 (1.71-2.43) | 2.31 (1.96-2.57) | **0.079** | ns | ns |  |
| GDNF | 2.26 (1.88-2.47) | 2.24 (2.03-2.46) | ns | 1.96 (1.68-2.21) | 2.15 (1.73-2.34) | **0.097** | 1.83 (1.58-2.06) | 1.88 (1.72-2.15) | ns | 1.67 (1.42-2) | 1.83 (1.59-2.07) | **0.078** | 1.87 (1.53-2.16) | 2 (1.69-2.3) | **0.085** | **0.063** | **0.089** |  |
| CDCP1 | 3.36 (3.03-3.6) | 3.29 (3.13-3.78) | ns | 3.68 (3.42-4.11) | 3.66 (3.32-3.86) | ns | 4.1 (3.75-4.34) | 3.94 (3.68-4.28) | ns | 4.5 (4.07-4.79) | 4.27 (3.98-4.88) | ns | 4.81 (4.37-5.2) | 4.66 (4.15-5.2) | ns | ns | ns |  |
| CD244 | 6.17 (5.95-6.44) | 6.23 (5.92-6.51) | ns | 6.05 (5.78-6.32) | 6 (5.75-6.33) | ns | 5.99 (5.74-6.27) | 5.93 (5.72-6.2) | ns | 6.03 (5.72-6.32) | 5.97 (5.81-6.3) | ns | 5.94 (5.55-6.1) | 5.85 (5.66-6.08) | ns | ns | ns |  |
| IL7 | 3.23 (2.81-3.55) | 3.23 (2.83-3.57) | ns | 3.3 (3-3.74) | 3.27 (2.78-3.52) | ns | 3.33 (3-3.66) | 3.2 (2.87-3.5) | **0.093** | 3.12 (2.79-3.49) | 3 (2.75-3.43) | ns | 3.24 (2.9-3.63) | 3.24 (2.99-3.64) | ns | ns | ns |  |
| OPG | 11.16 (10.8-11.49) | 11.14 (10.87-11.34) | ns | 11.27 (10.92-11.59) | 11.12 (10.87-11.41) | ns | 11.42 (11.11-11.75) | 11.33 (10.95-11.64) | ns | 11.72 (11.33-12.25) | 11.7 (11.22-11.87) | ns | 11.96 (11.64-12.46) | 11.86 (11.52-12.22) | ns | ns | ns |  |
| LAP TGF-beta-1 | 8.05 (7.67-8.42) | 8.03 (7.64-8.3) | ns | 7.82 (7.57-8.13) | 7.75 (7.43-8.16) | ns | 7.9 (7.48-8.11) | 7.95 (7.63-8.23) | ns | 8.1 (7.83-8.38) | 8.12 (7.89-8.38) | ns | 8.35 (8.04-8.73) | 8.48 (8.14-8.73) | ns | ns | ns |  |
| uPA | 10.39 (10.13-10.67) | 10.45 (10.19-10.75) | ns | 10.35 (10.06-10.59) | 10.36 (10.1-10.54) | ns | 10.54 (10.33-10.85) | 10.52 (10.29-10.81) | ns | 10.89 (10.54-11.35) | 10.94 (10.67-11.2) | ns | 11.35 (10.99-11.68) | 11.42 (11.17-11.68) | ns | ns | ns |  |
| IL6 | 2.99 (2.52-3.5) | 2.99 (2.67-3.46) | ns | 3.07 (2.58-3.6) | 3.01 (2.74-3.48) | ns | 2.84 (2.53-3.38) | 2.89 (2.45-3.36) | ns | 3.16 (2.8-3.63) | 3.18 (2.77-3.55) | ns | 3.32 (2.91-3.76) | 3.24 (2.99-3.57) | ns | ns | ns |  |
| IL-17C | 2.52 (2.21-2.93) | 2.72 (2.19-3.28) | ns | 2.78 (2.32-3.35) | 2.76 (2.11-3.19) | ns | 2.87 (2.39-3.35) | 3.04 (2.58-3.6) | **0.098** | 3.11 (2.62-3.53) | 2.97 (2.5-3.38) | ns | 2.97 (2.62-3.47) | 3.1 (2.6-3.5) | ns | ns | ns |  |
| MCP-1 | 11.64 (11.33-11.98) | 11.77 (11.4-11.92) | ns | 11.56 (11.29-11.8) | 11.48 (11.15-12.05) | ns | 11.41 (11.09-11.7) | 11.4 (11.13-11.73) | ns | 11.44 (11.07-11.8) | 11.37 (11.1-11.78) | ns | 11.34 (11.03-11.62) | 11.48 (11.09-11.66) | ns | ns | ns |  |
| IL-17A | 1.36 (0.98-1.84) | 1.28 (0.99-1.78) | ns | 1.35 (1.12-1.93) | 1.37 (0.94-1.71) | ns | 1.18 (0.85-1.75) | 1.2 (0.87-1.59) | ns | 1.27 (0.8-1.89) | 1.14 (0.82-1.42) | ns | 1.13 (0.81-1.46) | 1.11 (0.8-1.58) | ns | ns | ns |  |
| CXCL11 | 8.99 (8.49-9.57) | 8.89 (8.3-9.48) | ns | 9.04 (8.57-9.44) | 8.73 (8.1-9.37) | **0.072** | 9.02 (8.49-9.43) | 8.87 (8.18-9.55) | ns | 9.17 (8.67-9.82) | 9 (8.29-9.44) | **0.029** | 9.34 (8.8-9.93) | 9.06 (8.6-9.78) | ns | **0.032** | **0.045** |  |
| AXIN1 | 2.79 (2.25-3.32) | 2.93 (2.6-3.56) | ns | 2.61 (2.4-2.98) | 2.71 (2.46-3.38) | ns | 2.47 (2.06-2.8) | 2.71 (2.27-3.09) | **0.049** | 2.85 (2.39-3.38) | 3 (2.54-3.37) | ns | 2.46 (2-2.94) | 2.71 (2.21-3.15) | ns | ns | ns |  |
| TRAIL | 8.65 (8.4-8.91) | 8.74 (8.46-8.92) | ns | 8.51 (8.32-8.76) | 8.51 (8.29-8.83) | ns | 8.42 (8.14-8.65) | 8.43 (8.11-8.73) | ns | 8.43 (8.15-8.64) | 8.41 (8.06-8.64) | ns | 8.4 (8.08-8.71) | 8.35 (8.15-8.69) | ns | ns | ns |  |
| IL-20RA | 0.94 (0.65-1.21) | 0.86 (0.65-1.09) | ns | 0.87 (0.65-1.18) | 0.84 (0.65-1.09) | ns | 0.9 (0.66-1.13) | 0.92 (0.68-1.18) | ns | 0.97 (0.7-1.39) | 1.02 (0.82-1.24) | ns | 1.14 (0.75-1.47) | 1.3 (0.95-1.54) | ns | ns | ns |  |
| CXCL9 | 6.96 (6.6-7.32) | 6.91 (6.46-7.44) | ns | 6.94 (6.6-7.68) | 6.93 (6.54-7.56) | ns | 6.76 (6.44-7.34) | 6.82 (6.43-7.29) | ns | 6.91 (6.48-7.35) | 6.78 (6.46-7.29) | ns | 6.78 (6.41-7.15) | 6.68 (6.43-7.06) | ns | ns | ns |  |
| CST5 | 6.72 (6.4-6.96) | 6.79 (6.44-7.16) | ns | 6.75 (6.42-7.09) | 6.68 (6.31-7.08) | ns | 6.7 (6.46-6.94) | 6.76 (6.3-7.26) | ns | 6.58 (6.3-6.87) | 6.65 (6.25-7) | ns | 6.9 (6.55-7.14) | 6.95 (6.59-7.27) | ns | ns | ns |  |
| IL-2RB | 0.18 (0.18-0.18) | 0.18 (0.18-0.18) | ns | 0.18 (0.18-0.18) | 0.18 (0.18-0.18) | ns | 0.18 (0.18-0.18) | 0.18 (0.18-0.18) | ns | 0.18 (0.18-0.18) | 0.18 (0.18-0.18) | ns | 0.18 (0.18-0.18) | 0.18 (0.18-0.18) | ns | **0.082** | **ns** |  |
| IL-1 alpha | 0.3 (0.3-0.3) | 0.3 (0.3-0.3) | ns | 0.3 (0.3-0.3) | 0.3 (0.3-0.3) | ns | 0.3 (0.3-0.3) | 0.3 (0.3-0.3) | ns | 0.3 (0.3-0.3) | 0.3 (0.3-0.3) | **0.012** | 0.3 (0.3-0.3) | 0.3 (0.3-0.3) | ns | ns | ns |  |
| OSM | 4.76 (4.36-5.46) | 5.02 (4.38-5.55) | ns | 4.86 (4.48-5.57) | 4.95 (4.45-5.44) | ns | 4.97 (4.56-5.36) | 5 (4.67-5.62) | ns | 5.38 (4.93-5.74) | 5.49 (5.06-5.91) | ns | 5.04 (4.61-5.52) | 5.12 (4.81-5.6) | ns | ns | ns |  |
| IL2 | 1.2 (1.2-1.2) | 1.2 (1.2-1.2) | ns | 1.2 (1.2-1.2) | 1.2 (1.2-1.2) | ns | 1.2 (1.2-1.2) | 1.2 (1.2-1.2) | ns | 1.2 (1.2-1.2) | 1.2 (1.2-1.2) | ns | 1.2 (1.2-1.2) | 1.2 (1.2-1.2) | ns | ns | ns |  |
| CXCL1 | 9.83 (9.37-10.29) | 9.91 (9.34-10.56) | ns | 9.84 (9.39-10.3) | 9.88 (9.41-10.23) | ns | 9.97 (9.5-10.38) | 10.28 (9.69-10.82) | **0.039** | 10.29 (9.83-10.66) | 10.29 (9.66-10.74) | ns | 10.45 (10-10.7) | 10.5 (10.05-11.09) | ns | ns | ns |  |
| TSLP | 0.47 (0.47-0.47) | 0.47 (0.47-0.75) | **0.001** | 0.47 (0.47-0.49) | 0.47 (0.47-0.55) | ns | 0.47 (0.47-0.47) | 0.47 (0.47-0.47) | ns | 0.47 (0.47-0.48) | 0.47 (0.47-0.49) | ns | 0.47 (0.47-0.47) | 0.47 (0.47-0.5) | ns | ns | ns |  |
| CCL4 | 8.34 (7.7-8.74) | 8.73 (8.13-9.17) | **0.015** | 8.27 (7.89-8.69) | 8.45 (8.02-9.09) | ns | 8.27 (7.91-8.7) | 8.53 (8.28-8.98) | **0.018** | 8.42 (8-8.91) | 8.68 (8.1-9.04) | ns | 8.29 (7.72-8.73) | 8.46 (8.08-9.02) | **0.031** | **0.055** | **0.078** |  |
| CD6 | 4.75 (4.42-5.02) | 4.88 (4.42-5.26) | ns | 4.47 (4.19-4.86) | 4.58 (4.13-4.95) | ns | 4.39 (4.02-4.7) | 4.46 (3.99-4.66) | ns | 4.36 (3.9-4.74) | 4.28 (4-4.62) | ns | 4.22 (3.9-4.46) | 4.25 (3.88-4.64) | ns | ns | ns |  |
| SCF | 9.32 (9.07-9.57) | 9.32 (8.96-9.56) | ns | 9.07 (8.78-9.45) | 9.1 (8.86-9.37) | ns | 8.95 (8.68-9.17) | 9.01 (8.66-9.28) | ns | 8.84 (8.56-9.14) | 8.86 (8.46-9.07) | ns | 8.75 (8.38-9.08) | 8.76 (8.42-8.99) | ns | ns | ns |  |
| IL18 | 9.62 (9.28-10.05) | 9.55 (9.17-10.05) | ns | 9.74 (9.32-10.02) | 9.74 (9.32-10.09) | ns | 10.07 (9.67-10.45) | 10.11 (9.6-10.6) | ns | 10.24 (9.9-10.7) | 10.36 (10.08-10.69) | ns | 10.27 (9.82-10.61) | 10.4 (10.13-10.76) | **0.049** | ns | ns |  |
| SLAMF1 | 1.78 (1.57-2) | 1.84 (1.57-2.04) | ns | 1.66 (1.37-2) | 1.69 (1.39-1.92) | ns | 1.56 (1.41-1.84) | 1.62 (1.37-1.91) | ns | 1.52 (1.36-1.87) | 1.58 (1.42-1.85) | ns | 1.56 (1.36-1.81) | 1.57 (1.3-1.78) | ns | ns | ns |  |
| TGF-alpha | 4.11 (3.74-4.87) | 4.52 (3.69-4.89) | ns | 4.33 (3.71-4.78) | 4.36 (3.76-4.85) | ns | 4.13 (3.79-4.55) | 4.37 (3.88-4.66) | ns | 4.43 (4.13-4.75) | 4.46 (4.14-4.96) | ns | 4.29 (3.93-4.73) | 4.41 (3.98-4.84) | ns | ns | ns |  |
| MCP-4 | 14.61 (14.14-15.08) | 14.62 (14.14-15.06) | ns | 14.4 (13.99-14.79) | 14.52 (13.7-14.76) | ns | 14.21 (13.72-14.64) | 14.29 (13.87-14.68) | ns | 14.09 (13.61-14.47) | 13.92 (13.56-14.3) | ns | 13.91 (13.42-14.33) | 13.82 (13.49-14.18) | ns | ns | ns |  |
| CCL11 | 7.77 (7.36-8.05) | 7.82 (7.46-8.02) | ns | 7.34 (7.06-7.8) | 7.3 (7.04-7.76) | ns | 7.12 (6.83-7.36) | 7.24 (6.86-7.52) | ns | 6.93 (6.55-7.25) | 7.06 (6.7-7.3) | ns | 7.09 (6.7-7.36) | 7.15 (6.74-7.32) | ns | ns | ns |  |
| TNFSF14 | 6.6 (6.08-7.52) | 6.82 (6.26-7.4) | ns | 6.41 (5.99-7.12) | 6.63 (6.06-7.37) | ns | 6.45 (5.87-7.07) | 6.55 (6.06-7.13) | ns | 7.17 (6.68-7.5) | 7.22 (6.69-7.79) | ns | 6.85 (6.17-7.38) | 6.73 (6.33-7.28) | ns | ns | ns |  |
| FGF-23 | 0.92 (0.71-1.24) | 1.01 (0.7-1.21) | ns | 0.94 (0.69-1.23) | 0.95 (0.71-1.17) | ns | 1.02 (0.75-1.32) | 1.03 (0.78-1.43) | ns | 1.3 (1.03-1.66) | 1.24 (1.02-1.54) | ns | 1.62 (1.31-2.32) | 1.56 (1.17-2.17) | ns | ns | ns |  |
| IL-10RA | 1.35 (1.03-1.66) | 1.5 (1.16-2.08) | **0.087** | 1.29 (0.82-1.78) | 1.35 (1-2.11) | ns | 1.16 (0.87-1.5) | 1.28 (0.95-1.79) | ns | 1.21 (0.96-1.57) | 1.25 (0.99-1.92) | ns | 1.14 (0.82-1.59) | 1.24 (0.96-1.62) | ns | ns | ns |  |
| FGF-5 | 1.84 (1.68-2.06) | 1.9 (1.77-2.08) | ns | 1.82 (1.56-2.02) | 1.83 (1.64-2.03) | ns | 1.68 (1.49-1.86) | 1.76 (1.65-1.9) | ns | 1.71 (1.56-1.9) | 1.78 (1.59-1.91) | ns | 1.72 (1.48-1.98) | 1.77 (1.62-1.93) | ns | ns | ns |  |
| MMP-1 | 16.73 (16.14-17.1) | 16.45 (15.8-16.88) | **0.035** | 16.58 (15.8-16.96) | 16.27 (15.54-16.74) | **0.092** | 16.73 (16-17.15) | 16.35 (15.88-16.98) | **0.067** | 17.11 (16.5-17.43) | 16.7 (16.29-17.07) | **0.012** | 17.18 (16.75-17.61) | 16.97 (16.44-17.37) | **0.069** | **0.099** | **ns** |  |
| LIF-R | 4.97 (4.56-5.27) | 5.06 (4.74-5.29) | ns | 6.1 (5.69-6.65) | 6.01 (5.76-6.52) | ns | 6.63 (6.35-6.97) | 6.67 (6.35-7.17) | ns | 7.03 (6.67-7.48) | 6.93 (6.44-7.39) | ns | 7.41 (7.07-7.76) | 7.35 (6.79-7.91) | ns | ns | ns |  |
| FGF-21 | 4.82 (4.29-5.53) | 4.86 (3.95-5.89) | ns | 4.6 (3.79-5.73) | 4.63 (3.5-5.33) | ns | 4.68 (4.07-5.63) | 4.35 (3.52-5.46) | ns | 5.41 (4.26-6.75) | 5.06 (4.48-6.3) | ns | 5.99 (5.14-7.11) | 5.4 (4.38-6.91) | **0.058** | ns | ns |  |
| CCL19 | 10.46 (10.08-11.06) | 10.62 (10.2-11.07) | ns | 10.3 (10.04-10.66) | 10.43 (10.07-11.01) | ns | 10.13 (9.68-10.62) | 10.23 (9.86-10.63) | ns | 10.04 (9.57-10.75) | 10.1 (9.76-10.69) | ns | 9.95 (9.62-10.73) | 10.11 (9.83-10.6) | ns | ns | ns |  |
| IL-15RA | 2.33 (2.04-2.45) | 2.34 (2.06-2.54) | ns | 2.19 (2.03-2.46) | 2.21 (2.07-2.46) | ns | 2.29 (2.08-2.46) | 2.41 (2.17-2.62) | **0.096** | 2.4 (2.24-2.67) | 2.49 (2.26-2.68) | ns | 2.41 (2.17-2.69) | 2.49 (2.31-2.69) | ns | ns | ns |  |
| IL-10RB | 7.89 (7.71-7.99) | 7.96 (7.68-8.17) | ns | 7.94 (7.67-8.25) | 7.94 (7.7-8.2) | ns | 8.12 (7.94-8.4) | 8.22 (7.92-8.49) | ns | 8.4 (8.13-8.7) | 8.48 (8.2-8.66) | ns | 8.45 (8.26-8.69) | 8.53 (8.36-8.7) | ns | ns | ns |  |
| IL-22 RA1 | 1.56 (1.56-1.56) | 1.56 (1.56-1.81) | **0.061** | 1.56 (1.56-1.56) | 1.56 (1.56-1.7) | ns | 1.56 (1.56-1.75) | 1.56 (1.56-1.96) | ns | 1.56 (1.56-1.85) | 1.56 (1.56-1.85) | ns | 1.56 (1.56-1.76) | 1.56 (1.56-1.87) | ns | ns | ns |  |
| IL-18R1 | 9.52 (9.29-9.77) | 9.43 (9.18-9.88) | ns | 9.53 (9.25-9.8) | 9.46 (9.11-9.83) | ns | 9.61 (9.25-9.77) | 9.44 (9.16-9.82) | ns | 9.71 (9.29-9.99) | 9.69 (9.48-9.86) | ns | 9.82 (9.42-10.05) | 9.71 (9.52-10.06) | ns | ns | ns |  |
| PD-L1 | 6.14 (5.91-6.55) | 6.22 (5.98-6.53) | ns | 7.01 (6.55-7.27) | 6.84 (6.56-7.1) | ns | 7.84 (7.62-8.09) | 7.87 (7.55-8.1) | ns | 8.58 (8.15-9.02) | 8.5 (8.22-8.9) | ns | 8.86 (8.56-9.16) | 8.84 (8.55-9.26) | ns | ns | ns |  |
| Beta-NGF | 2.18 (2.18-2.18) | 2.18 (2.18-2.18) | ns | 2.18 (2.18-2.18) | 2.18 (2.18-2.18) | ns | 2.18 (2.18-2.18) | 2.18 (2.18-2.18) | ns | 2.18 (2.18-2.18) | 2.18 (2.18-2.18) | ns | 2.18 (2.18-2.18) | 2.18 (2.18-2.18) | ns | ns | ns |  |
| CXCL5 | 13.18 (12.44-13.64) | 12.98 (12.28-13.49) | ns | 12.91 (12.54-13.6) | 12.83 (12.27-13.46) | ns | 12.97 (12.38-13.55) | 13.02 (12.38-13.37) | ns | 12.68 (12.07-13.19) | 12.35 (11.76-13.01) | ns | 12.71 (11.91-13.37) | 12.51 (12-12.95) | ns | **0.075** | **0.098** |  |
| TRANCE | 4.43 (4.05-5.12) | 4.63 (4.13-5.18) | ns | 4.03 (3.61-4.42) | 4.03 (3.69-4.46) | ns | 3.62 (3.24-3.96) | 3.71 (3.42-4.05) | ns | 3.44 (3.06-3.81) | 3.55 (3.23-3.74) | ns | 3.15 (2.86-3.63) | 3.31 (2.98-3.56) | ns | ns | ns |  |
| HGF | 9.3 (9.01-9.81) | 9.43 (9.08-9.79) | ns | 9.38 (9.05-9.79) | 9.39 (9.02-9.65) | ns | 9.33 (9.07-9.71) | 9.35 (9.18-9.67) | ns | 9.66 (9.37-9.92) | 9.59 (9.37-10.01) | ns | 9.66 (9.43-10.03) | 9.61 (9.39-9.99) | ns | ns | ns |  |
| IL-12B | 6.44 (6.08-6.84) | 6.52 (6.02-6.87) | ns | 6.14 (5.84-6.68) | 6.2 (5.83-6.57) | ns | 5.95 (5.49-6.4) | 5.92 (5.53-6.3) | ns | 5.8 (5.24-6.17) | 5.64 (5.32-6.1) | ns | 5.64 (5.12-6.1) | 5.72 (5.34-5.99) | ns | ns | ns |  |
| IL-24 | 2.1 (2.1-2.1) | 2.1 (2.1-2.1) | ns | 2.1 (2.1-2.1) | 2.1 (2.1-2.1) | ns | 2.1 (2.1-2.1) | 2.1 (2.1-2.1) | ns | 2.1 (2.1-2.1) | 2.1 (2.1-2.1) | ns | 2.1 (2.1-2.26) | 2.1 (2.1-2.17) | ns | ns | ns |  |
| IL13 | 0.8 (0.8-1.24) | 0.81 (0.8-1.13) | ns | 0.8 (0.8-1.05) | 0.8 (0.8-1.04) | ns | 0.8 (0.8-1.06) | 0.8 (0.8-1.13) | ns | 0.8 (0.8-0.91) | 0.8 (0.8-0.8) | ns | 0.8 (0.8-0.8) | 0.8 (0.8-1.08) | ns | ns | ns |  |
| ARTN | 0.41 (0.41-0.65) | 0.55 (0.41-0.8) | **0.037** | 0.47 (0.41-0.67) | 0.54 (0.41-0.75) | ns | 0.69 (0.46-0.98) | 0.84 (0.63-1.12) | **0.013** | 1.33 (0.99-1.6) | 1.36 (1.15-1.81) | ns | 1.92 (1.37-2.44) | 1.93 (1.69-2.38) | ns | **0.035** | **0.049** |  |
| MMP-10 | 9.43 (9.14-9.91) | 9.43 (9.18-9.87) | ns | 9.51 (9.19-9.82) | 9.55 (9.23-9.87) | ns | 9.4 (9.13-9.85) | 9.47 (9.02-9.8) | ns | 9.36 (9-9.75) | 9.4 (9.01-9.65) | ns | 9.34 (9.04-9.66) | 9.26 (8.93-9.53) | ns | ns | ns |  |
| IL10 | 3.53 (3.19-3.76) | 3.73 (3.28-4.01) | **0.064** | 3.5 (3.24-3.88) | 3.6 (3.29-4.09) | ns | 3.53 (3.24-3.91) | 3.63 (3.31-4.09) | ns | 3.56 (3.22-3.8) | 3.68 (3.24-4.05) | ns | 3.33 (3.08-3.74) | 3.61 (3.29-3.8) | **0.031** | ns | ns |  |
| TNF | 3.34 (2.93-3.69) | 3.33 (2.98-3.72) | ns | 3.2 (2.82-3.65) | 3.26 (2.98-3.68) | ns | 3.24 (2.84-3.61) | 3.3 (3.1-3.51) | ns | 3.3 (2.61-3.78) | 3.39 (3.06-3.77) | ns | 3.38 (2.96-3.74) | 3.49 (3.25-3.8) | ns | ns | ns |  |
| CCL23 | 10.85 (10.6-11.18) | 10.78 (10.38-11.12) | ns | 10.56 (10.31-11.08) | 10.63 (10.29-10.94) | ns | 10.48 (10.24-10.81) | 10.49 (10.18-10.73) | ns | 10.37 (10.14-10.75) | 10.48 (10.07-10.76) | ns | 10.2 (9.92-10.53) | 10.22 (10-10.52) | ns | ns | ns |  |
| CD5 | 5.72 (5.45-5.9) | 5.74 (5.38-6.13) | ns | 5.48 (5.27-5.9) | 5.6 (5.16-5.95) | ns | 5.36 (5.21-5.7) | 5.41 (5.17-5.79) | ns | 5.35 (5.03-5.73) | 5.41 (4.99-5.61) | ns | 5.19 (4.94-5.5) | 5.15 (4.94-5.68) | ns | ns | ns |  |
| CCL3 | 5.91 (5.56-6.34) | 6.13 (5.77-6.6) | **0.068** | 5.93 (5.6-6.3) | 6 (5.56-6.52) | ns | 6.11 (5.66-6.48) | 6.18 (5.84-6.55) | ns | 6.35 (6.02-6.65) | 6.53 (6.13-6.93) | ns | 6.14 (5.78-6.51) | 6.34 (5.88-6.63) | ns | ns | ns |  |
| Flt3L | 10.04 (9.86-10.31) | 10.16 (9.86-10.43) | ns | 10.18 (10.03-10.41) | 10.22 (9.9-10.6) | ns | 10.21 (10.06-10.47) | 10.46 (10.01-10.63) | ns | 10.32 (10.05-10.58) | 10.41 (10.13-10.61) | ns | 10.28 (10.06-10.53) | 10.27 (10.11-10.71) | ns | ns | ns |  |
| CXCL6 | 9.16 (8.42-9.64) | 9.27 (8.78-9.69) | ns | 9.07 (8.59-9.4) | 9.09 (8.49-9.53) | ns | 8.93 (8.56-9.63) | 9.25 (8.67-9.79) | ns | 9.09 (8.59-9.41) | 8.96 (8.6-9.33) | ns | 9.3 (8.69-9.7) | 9.22 (8.81-9.54) | ns | ns | ns |  |
| CXCL10 | 0.34 (0.34-0.51) | 0.34 (0.34-0.53) | ns | 0.34 (0.34-0.47) | 0.34 (0.34-0.61) | ns | 0.34 (0.34-0.51) | 0.34 (0.34-0.47) | ns | 0.34 (0.34-0.52) | 0.34 (0.34-0.51) | ns | 0.34 (0.34-0.51) | 0.35 (0.34-0.59) | ns | ns | ns |  |
| 4E-BP1 | 6.25 (5.47-6.98) | 6.22 (5.59-6.88) | ns | 6.1 (5.31-6.84) | 6.1 (5.59-6.73) | ns | 5.75 (4.12-6.48) | 6.22 (5.39-6.7) | **0.037** | 5.47 (3.55-6.6) | 5.89 (4.93-6.92) | **0.032** | 5.57 (3.85-6.3) | 5.65 (4.87-6.32) | ns | ns | ns |  |
| IL-20 | 1.24 (1.24-1.43) | 1.24 (1.24-1.38) | ns | 1.24 (1.24-1.42) | 1.24 (1.24-1.38) | ns | 1.24 (1.24-1.34) | 1.24 (1.24-1.36) | ns | 1.24 (1.24-1.42) | 1.24 (1.24-1.36) | ns | 1.24 (1.24-1.43) | 1.24 (1.24-1.4) | ns | ns | ns |  |
| SIRT2 | 3.14 (2.58-3.88) | 3.32 (2.82-3.78) | ns | 3.1 (2.73-3.72) | 3.12 (2.67-4.1) | ns | 2.79 (2.44-3.39) | 3.09 (2.65-3.62) | **0.081** | 3.3 (2.68-3.99) | 3.38 (2.92-4.54) | ns | 2.87 (2.51-3.45) | 2.97 (2.48-3.39) | ns | ns | ns |  |
| CCL28 | 3.2 (2.76-3.57) | 3.45 (2.86-4.02) | ns | 3.88 (3.19-4.45) | 3.85 (3.19-4.59) | ns | 5.23 (4.4-5.98) | 5.38 (4.6-6.03) | ns | 5.91 (5.24-6.35) | 5.9 (5.34-6.29) | ns | 5.86 (5.34-6.4) | 6.04 (5.5-6.4) | ns | ns | ns |  |
| DNER | 9.69 (9.47-10.01) | 9.85 (9.52-10.11) | ns | 9.71 (9.41-9.96) | 9.75 (9.4-9.99) | ns | 9.6 (9.3-9.77) | 9.59 (9.24-9.84) | ns | 9.52 (9.21-9.78) | 9.52 (9.25-9.85) | ns | 9.5 (9.17-9.71) | 9.47 (9.29-9.87) | ns | ns | ns |  |
| EN-RAGE | 3.76 (3.4-4.34) | 4.09 (3.42-4.61) | **0.087** | 4.06 (3.67-4.55) | 4.24 (3.66-4.61) | ns | 3.58 (3.25-4.01) | 3.73 (3.38-4.33) | **0.086** | 3.68 (3.08-4.2) | 4 (3.38-4.54) | **0.05** | 3.57 (2.97-4) | 3.47 (3.02-4.21) | ns | **0.032** | **0.045** |  |
| CD40 | 11.37 (11.03-11.56) | 11.43 (11.11-11.73) | ns | 11.15 (10.93-11.44) | 11.28 (10.95-11.6) | ns | 11.08 (10.82-11.42) | 11.21 (10.96-11.42) | ns | 11.29 (10.98-11.51) | 11.23 (11.05-11.6) | ns | 11.21 (10.92-11.48) | 11.19 (11-11.47) | ns | ns | ns |  |
| IL33 | 2.12 (2.12-2.12) | 2.12 (2.12-2.12) | ns | 2.12 (2.12-2.12) | 2.12 (2.12-2.12) | ns | 2.12 (2.12-2.12) | 2.12 (2.12-2.12) | ns | 2.12 (2.12-2.12) | 2.12 (2.12-2.12) | ns | 2.12 (2.12-2.12) | 2.12 (2.12-2.12) | ns | ns | ns |  |
| IFN-gamma | 6.23 (5.72-6.9) | 6.15 (5.62-7) | ns | 6.47 (5.88-6.97) | 6.19 (5.72-6.77) | ns | 6.27 (5.81-6.88) | 6.16 (5.67-6.93) | ns | 6.29 (5.76-7.18) | 6.23 (5.71-6.97) | ns | 6.02 (5.52-6.44) | 5.8 (5.27-6.4) | ns | ns | ns |  |
| FGF-19 | 8.69 (8.15-9.4) | 9.17 (8.31-9.59) | ns | 8.82 (8.02-9.44) | 8.85 (8.14-9.44) | ns | 8.65 (8.31-9.53) | 9.05 (8.51-9.68) | ns | 8.32 (7.5-8.8) | 8.25 (7.79-8.9) | ns | 8.45 (7.83-9.31) | 8.67 (7.73-9.43) | ns | ns | ns |  |
| IL4 | 0.88 (0.48-1.38) | 1.02 (0.57-1.49) | ns | 0.84 (0.48-1.26) | 1.1 (0.48-1.62) | ns | 0.74 (0.48-1.29) | 1 (0.48-1.58) | ns | 0.81 (0.48-1.35) | 1.03 (0.48-1.9) | ns | 0.87 (0.48-1.29) | 1.01 (0.48-1.84) | **0.081** | **0.085** | **ns** |  |
| LIF | 0.6 (0.6-0.7) | 0.6 (0.6-0.83) | ns | 0.6 (0.6-0.76) | 0.6 (0.6-0.73) | ns | 0.6 (0.6-0.69) | 0.6 (0.6-0.74) | ns | 0.6 (0.6-0.69) | 0.6 (0.6-0.7) | ns | 0.6 (0.6-0.73) | 0.6 (0.6-0.72) | ns | ns | ns |  |
| NRTN | 0.59 (0.56-0.94) | 0.65 (0.56-0.94) | ns | 0.56 (0.56-1.04) | 0.61 (0.56-0.83) | ns | 0.56 (0.56-0.79) | 0.56 (0.56-0.85) | ns | 0.56 (0.56-0.89) | 0.59 (0.56-0.85) | ns | 0.58 (0.56-0.84) | 0.59 (0.56-0.76) | ns | ns | ns |  |
| MCP-2 | 9.98 (9.48-10.5) | 9.88 (9.39-10.47) | ns | 9.79 (9.25-10.24) | 9.68 (9.03-10.19) | ns | 9.51 (8.96-10.01) | 9.47 (8.96-10.06) | ns | 9.32 (8.94-9.82) | 9.32 (8.8-9.81) | ns | 9.26 (8.78-9.57) | 9.17 (8.56-9.41) | ns | ns | ns |  |
| CASP-8 | 2.35 (1.99-2.74) | 2.36 (2.04-2.88) | ns | 2.29 (1.99-2.75) | 2.28 (2.02-2.64) | ns | 2.22 (1.86-2.7) | 2.21 (1.94-2.6) | ns | 2.79 (2.55-3.45) | 2.91 (2.32-3.63) | ns | 2.31 (1.99-2.67) | 2.25 (1.87-2.48) | ns | ns | ns |  |
| CCL25 | 6.21 (5.87-6.46) | 6.23 (5.83-6.56) | ns | 6.1 (5.78-6.43) | 6.04 (5.63-6.44) | ns | 6.11 (5.86-6.44) | 6.03 (5.7-6.63) | ns | 6.15 (5.8-6.55) | 6.18 (5.71-6.69) | ns | 6.27 (5.95-6.57) | 6.18 (5.91-6.72) | ns | ns | ns |  |
| CX3CL1 | 3.65 (3.3-3.9) | 3.67 (3.32-4.05) | ns | 3.58 (3.14-3.9) | 3.64 (3.25-3.95) | ns | 3.59 (3.27-3.9) | 3.59 (3.26-3.83) | ns | 3.58 (3.3-3.82) | 3.63 (3.3-3.82) | ns | 3.61 (3.25-3.79) | 3.49 (3.23-3.9) | ns | ns | ns |  |
| TNFRSF9 | 5.19 (4.99-5.47) | 5.2 (4.95-5.5) | ns | 5.12 (4.91-5.39) | 5.13 (4.89-5.3) | ns | 4.97 (4.7-5.14) | 4.96 (4.63-5.23) | ns | 5.1 (4.71-5.3) | 4.99 (4.78-5.2) | ns | 4.87 (4.55-5.1) | 4.86 (4.63-5.24) | ns | ns | ns |  |
| NT-3 | 3.01 (2.69-3.25) | 3 (2.81-3.29) | ns | 2.94 (2.55-3.09) | 2.93 (2.67-3.19) | ns | 2.84 (2.6-2.98) | 2.95 (2.64-3.14) | **0.074** | 2.67 (2.47-2.91) | 2.71 (2.51-3.02) | ns | 2.75 (2.43-3.09) | 2.72 (2.48-2.87) | ns | **0.032** | **0.048** |  |
| TWEAK | 9.13 (8.86-9.39) | 9.07 (8.84-9.35) | ns | 8.83 (8.55-9.16) | 8.85 (8.57-9.13) | ns | 8.54 (8.32-8.82) | 8.55 (8.27-8.77) | ns | 8.43 (8.13-8.67) | 8.44 (8.06-8.69) | ns | 8.17 (7.97-8.63) | 8.32 (8.09-8.51) | ns | ns | ns |  |
| CCL20 | 7.19 (6.73-7.81) | 7.09 (6.69-7.87) | ns | 7.26 (6.43-7.83) | 6.84 (6.32-7.52) | **0.083** | 6.87 (6.49-7.44) | 6.89 (6.44-7.24) | ns | 7.1 (6.44-7.91) | 6.78 (6.36-7.32) | ns | 7.05 (6.48-7.56) | 6.94 (6.47-7.54) | ns | ns | ns |  |
| ST1A1 | 8.49 (7.48-9.63) | 8.61 (7.87-9.56) | ns | 8.1 (7.5-9.36) | 8.49 (7.49-9.63) | ns | 8.29 (7.37-9.21) | 8.29 (7.66-9.61) | ns | 9.25 (8.55-10.12) | 9.31 (8.49-10.13) | ns | 8.59 (7.77-9.17) | 8.27 (7.77-9.38) | ns | ns | ns |  |
| STAMBP | 5.38 (4.85-5.76) | 5.43 (4.99-5.85) | ns | 5.27 (4.97-5.65) | 5.26 (4.87-5.71) | ns | 5.04 (4.64-5.28) | 5.22 (4.94-5.56) | **0.023** | 5.15 (4.83-5.51) | 5.37 (4.95-6.08) | ns | 4.95 (4.71-5.4) | 5.19 (4.85-5.52) | ns | ns | ns |  |
| IL5 | 0.95 (0.87-1.68) | 1.15 (0.87-2.25) | ns | 0.88 (0.87-1.64) | 1.09 (0.87-2.69) | ns | 0.87 (0.87-1.59) | 1.2 (0.87-1.97) | **0.061** | 0.87 (0.87-1.48) | 1.15 (0.87-1.94) | **0.048** | 0.87 (0.87-1.62) | 0.87 (0.87-1.78) | ns | **0.079** | **0.098** |  |
| ADA | 5.44 (5.12-5.76) | 5.49 (5.19-5.73) | ns | 5.42 (5.14-5.67) | 5.39 (5.11-5.61) | ns | 5.25 (5.01-5.52) | 5.31 (5.14-5.62) | ns | 5.24 (4.99-5.46) | 5.39 (5.1-5.82) | **0.033** | 5.35 (5-5.58) | 5.36 (5.13-5.57) | ns | ns | ns |  |
| TNFB | 5.16 (4.96-5.41) | 5.2 (4.95-5.52) | ns | 5.06 (4.76-5.39) | 5.16 (4.78-5.41) | ns | 4.92 (4.71-5.2) | 4.99 (4.67-5.37) | ns | 4.99 (4.68-5.23) | 4.97 (4.71-5.29) | ns | 4.85 (4.62-5.12) | 4.9 (4.71-5.18) | ns | ns | ns |  |
| CSF-1 | 10.92 (10.65-11.16) | 10.89 (10.65-11.17) | ns | 11.04 (10.83-11.24) | 11.07 (10.71-11.31) | ns | 11.19 (11.02-11.37) | 11.22 (10.96-11.39) | ns | 11.21 (11.03-11.44) | 11.3 (10.99-11.56) | ns | 11.27 (11.03-11.45) | 11.33 (11.12-11.54) | ns | ns | ns |  |

Data shown are the normalized protein expression (NPX) values and are presented as median (interquartile range: 25^th^ – 75^th^ percentile). Statistical analyses were conducted between groups at specific time points using the Mann-Whitney U test. Bolded values represent statistical analyses with *p* values of <0.1. Longitudinal analyses across time points were performed using a linear mixed-effects model adjusting for maternal history of allergy (prenatal period), with multiple testing controlled using a false discovery rate (FDR) of 10%.

**Table S8:** Differential gut bacteria between control and AD infants which were identified at week 3, month 3 and month 6 of age and perturbed stool metabolites between control and AD infants at month 3 of age.

| **Species** | **Week 3** | | | **Month 3** | | | | **Month 6** | | | **Longitudinal *p*** | **FDR adjusted longitudinal *p*** | |
| --- | --- | --- | --- | --- | --- | --- | --- | --- | --- | --- | --- | --- | --- |
|  | **Control** | **AD** | ***p*** | **Control** | **AD** | | ***p*** | **Control** | **AD** | ***p*** |  |  |  |
| *Escherichia coli* | 101,815 ± 101,344 | 176,153 ± 177,998 | **0.037** | 111,339 ± 89,326 | 176,269 ± 13,292 | | **0.039** | 95,636 ± 87,007 | 101,985 ± 88,522 | ns | **0.028** | **0.035** |  |
| *Klebsiella pneumoniae* | 217,226 ± 162,969 | 327,410 ± 251,958 | **0.042** | 69,477 ± 55468 | 135,599 ± 133,835 | | **0.026** | 26,641 ± 24,577 | 31,417± 27,767 | **0.050** | **0.038** | **0.048** |  |
| *Bacteroides fragilis* | 64,379 ± 22,992 | 4,342 ± 3,784 | **0.037** | 184,664 ± 117,485 | 62,073 ± 53,013 | | **0.043** | 121,024 ± 103,117 | 41,116 ± 33,162 | **0.029** | **0.036** | **0.048** |  |
| *Bacteroides stercoris* | 22,251 ± 19,156 | 530 ± 823 | ns | 12,574 ± 11,449 | 147 ± 392 | | **0.030** | 6,235 ± 4,844 | 330 ± 816 | **0.030** | **0.025** | **0.035** |  |
| *Prevotella copri* | 502 ± 1,179 | 609 ± 1,235 | ns | 436 ± 983 | 87 ± 185 | | ns | 313 ± 612 | 98 ± 225 | ns | **0.045** | ns |  |
| *Bacteroides uniformis* | 5,716 ± 4,476 | 19,316 ± 10,296 | ns | 5,310 ± 4,690 | 696 ± 1,455 | | **0.018** | 5,259 ± 5,408 | 1,525 ± 1,720 | **0.029** | **0.020** | **0.028** |  |
| *Bacteroides thetaiotaomicron* | 29,706 ± 8,049 | 1,722 ± 1,234 | ns | 14,503 ± 17,409 | 1,037 ± 1,400 | | **0.038** | 7,687 ± 7,620 | 1,852 ± 1,385 | ns | **0.033** | **0.048** |  |
| *Alistipes putredinis* | 44 ± 111 | 16 ± 46 | ns | 39 ± 66 | 3.15 ± 11 | | ns | 148 ± 539 | 10 ± 35 | **0.035** | **0.032** | **0.048** |  |
| *Bacteroides eggerthii* | 363 ± 526 | 512 ± 1,233 | ns | 377± 724 | 229 ± 706 | | ns | 694 ± 126 | 120 ± 392 | **0.025** | **0.027** | **0.035** |  |
| *Parabacteroides merdae* | 449 ± 779 | 2,103 ± 2,607 | ns | 2,205 ± 2,683 | 400 ± 1,243 | | **0.011** | 9,682 ± 3,880 | 344 ± 295 | **0.029** | **0.024** | **0.035** |  |
| *Bacteroides ovatus* | 4,085 ± 2,063 | 8,069 ± 2,386 | ns | 5,265 ± 4,957 | 464 ± 447 | | ns | 87,04 ± 5,970 | 1,112 ± 1,609 | **0.025** | **0.023** | **0.035** |  |
| *Faecalibacterium prausnitzii* | 1,032 ± 1,659 | 200 ± 242 | **0.036** | 285 ± 304 | 276 ± 276 | | ns | 1,859± 1,326 | 374 ± 445 | **0.045** | **0.038** | **0.048** |  |
|  |  |  |  |  |  | |  |  |  |  |  |  |  |
| **Stool metabolites** | **Month 3** | | | | | | | | | | ***p*** |  | |
|  | **Control** | | | | | **AD** | | | | |  |  | |
| Isovaleric acid | 30.3 (13.8-46.6) | | | | | 11.6 (0-23.1) | | | | | **0.018** |  | |
| 2-methylbutyric acid | 20.2 (3.85-35.6) | | | | | 6.35 (0-15.9) | | | | | **0.043** |  | |
| Isobutyric acid | 25.6 (10.7-48.9) | | | | | 3.90 (0.390-15.2) | | | | | **0.045** |  | |

Metagenomic data are normalized reads of bacteria and are presented as geometric mean **±** standard deviation (SD). Metabolomics data shown are concentrations of stool metabolites (µg/g stool) and are presented as median (interquartile range: 25^th^ – 75^th^ percentile). Statistical analyses were conducted between groups at specific time points using the Mann-Whitney U test. Longitudinal analyses across time points were performed using a linear mixed-effects model adjusting for maternal antibiotic use during pregnancy/labour and mode of delivery (postnatal period), with multiple testing controlled using a false discovery rate (FDR) of 10%.


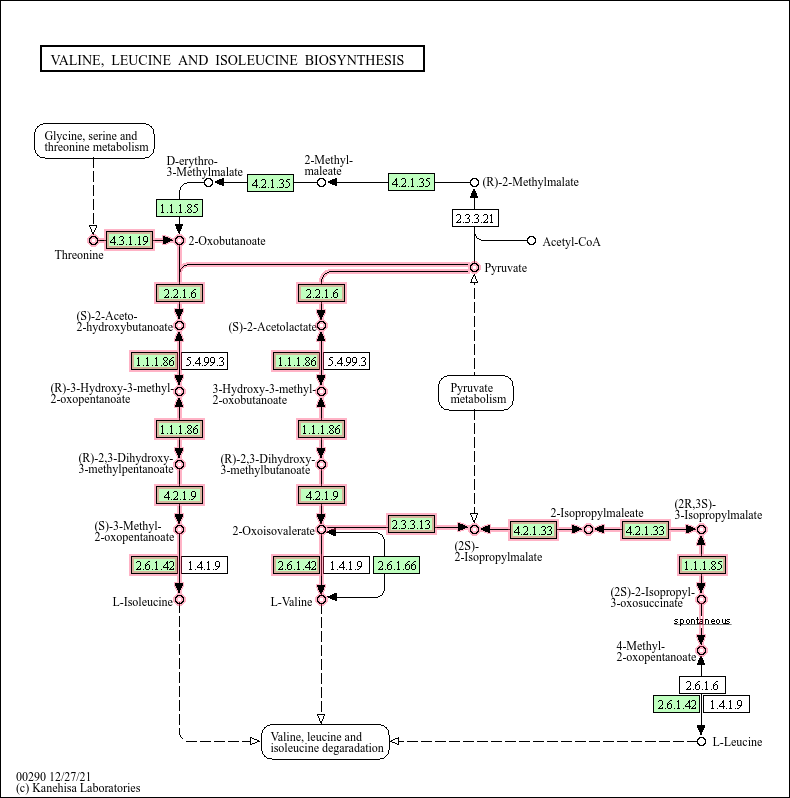

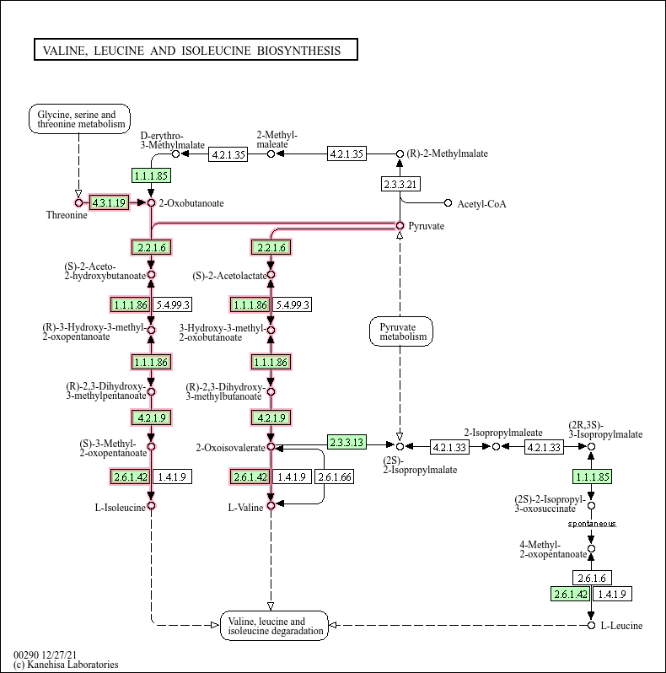


**Figure S1:** KEGG biosynthesis pathway for valine, leucine and isoleucine. (A) A schematic of the BCAA pathways annotated from the genomes of *Klebsiella pneumoniae* strains WHN643, WHN880 and WHN1212. (B) A schematic showing the BCAA pathway for *Roseburia intestinalis* DSM 14610^T^ (RefSeq no. GCF_000156535.1). The EC number of annotated enzymes are highlighted in green and red lines show adjoining annotated enzymes [15].

**
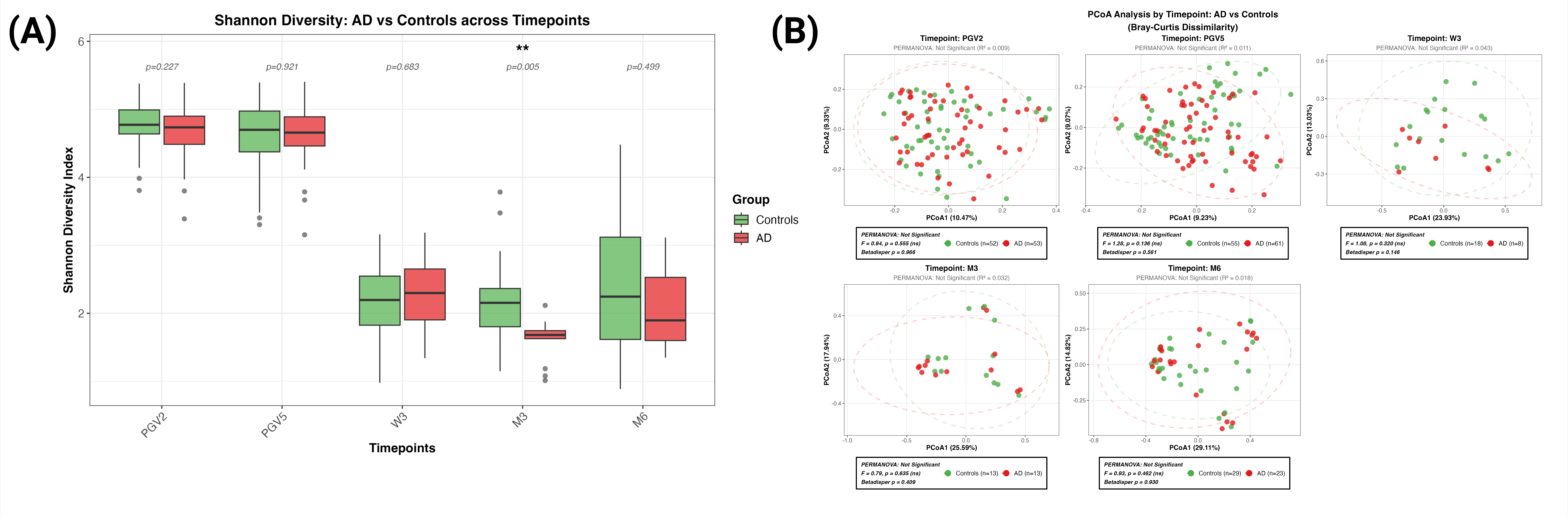
**

**Figure S2:** Community-level gut microbiome diversity across prenatal and postnatal timepoints. (A) Shannon α-diversity of maternal gut microbiomes during the first and third trimesters of pregnancy and of infant gut microbiomes at week 3, month 3, and month 6 of life between control and AD groups. (B) Principal coordinates analysis (PCoA) of Bray–Curtis β-diversity for the corresponding prenatal and postnatal timepoints between control and AD groups.


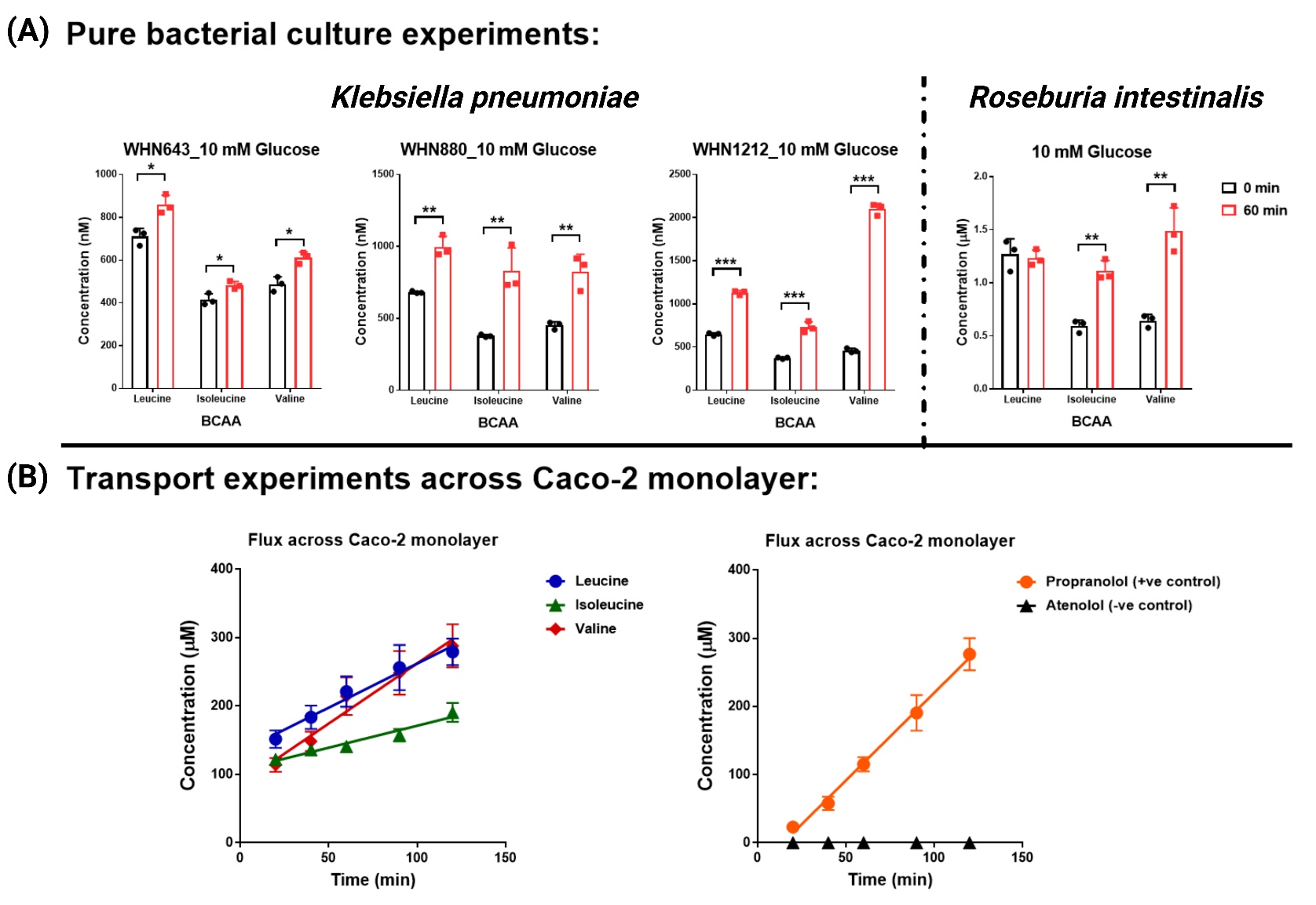


**Figure S3:** *In vitro* experiments to demonstrate (A) bacterial mediated synthesis of gut microbial-derived metabolites (branched chain amino acids – BCAA) into the culture media and (B) flux of BCAA across colonic lining from apical to basolateral compartment. (A) Pure bacterial culture experiments were performed as described in [3]. After incubation for 60 min in the presence of 10 mM glucose as substrate, *K. pneumoniae* synthesized and secreted all three BCAA into the culture media, while *R. intestinalis* only synthesized and secreted isoleucine and valine into the culture media. These results are congruent with the characterized functional metagenome of the bacterial strains where *K. pneumoniae* contained all enzymes necessary for synthesis of all three BCAA while *R. intestinalis* lacked 3-isopropylmalate dehydratase, which is integral for leucine biosynthesis. Multiple t-test with FDR correction by the Benjamini-Hochberg method was performed to identify statistically significant increases in specific BCAA levels across timepoints. *: *q*<0.05, **:*q<*0.01; ***: *q<*0.001 (B) *In vitro* Caco-2 transwell assay demonstrating flux of specific substrate (left – positive (propranolol) and negative (atenolol) control; right – BCAA) over time.

**References**

1. Duncan, S.H., et al., *Growth requirements and fermentation products of Fusobacterium prausnitzii, and a proposal to reclassify it as Faecalibacterium prausnitzii gen. nov., comb. nov.* Int J Syst Evol Microbiol, 2002. **52**(6): p. 2141-2146.

2. MacWilliams, M.P. and M.K. Liao, *Luria broth (LB) and Luria agar (LA) media and their uses protocol.* ASM MicrobeLibrary. American Society for Microbiology, 2006. **2006**: p. 1-4.

3. Li, T.-T., et al., *Microbiota metabolism of intestinal amino acids impacts host nutrient homeostasis and physiology.* Cell Host & Microbe, 2024. **32**(5): p. 661-675. e10.

4. Low, A., et al., *Complete genome sequence of Paramuribaculum intestinale DSM 100749(T), isolated from feces of C57BL/6 laboratory mice.* Microbiol Resour Announc, 2023. **12**(11): p. e0079723.

5. Chen, S., et al., *fastp: an ultra-fast all-in-one FASTQ preprocessor.* Bioinformatics, 2018. **34**(17): p. i884-i890.

6. Prjibelski, A., et al., *Using SPAdes De Novo Assembler.* Curr Protoc Bioinformatics, 2020. **70**(1): p. e102.

7. Galaxy, C., *The Galaxy platform for accessible, reproducible, and collaborative data analyses: 2024 update.* Nucleic Acids Res, 2024. **52**(W1): p. W83-W94.

8. Astashyn, A., et al., *Rapid and sensitive detection of genome contamination at scale with FCS-GX.* Genome Biol, 2024. **25**(1): p. 60.

9. Seemann, T., *Prokka: rapid prokaryotic genome annotation.* Bioinformatics, 2014. **30**(14): p. 2068-9.

10. Parks, D.H., et al., *CheckM: assessing the quality of microbial genomes recovered from isolates, single cells, and metagenomes.* Genome Res, 2015. **25**(7): p. 1043-1055.

11. Larsen, M.V., et al., *Multilocus sequence typing of total-genome-sequenced bacteria.* J Clin Microbiol, 2012. **50**(4): p. 1355-61.

12. Meier-Kolthoff, J.P., et al., *TYGS and LPSN: a database tandem for fast and reliable genome-based classification and nomenclature of prokaryotes.* Nucleic Acids Res, 2022. **50**(D1): p. D801-D807.

13. Meier-Kolthoff, J.P., et al., *Genome sequence-based species delimitation with confidence intervals and improved distance functions.* BMC Bioinformatics, 2013. **14**: p. 60.

14. Sayers, E.W., et al., *Database resources of the National Center for Biotechnology Information in 2025.* Nucleic Acids Res, 2024.

15. Kanehisa, M., Y. Sato, and K. Morishima, *BlastKOALA and GhostKOALA: KEGG tools for functional characterization of genome and metagenome sequences.* J Mol Biol, 2016. **428**(4): p. 726-731.

16. Van Breemen, R.B. and Y. Li, *Caco-2 cell permeability assays to measure drug absorption.* Expert opinion on drug metabolism & toxicology, 2005. **1**(2): p. 175-185.

17. Johnson, R.D. and R.J. Lewis, *Simultaneous Quantitation of Atenolol, Metoprolol, and Propranolol in Biological Matrices Via LC/MS*. 2005, United States. Department of Transportation. Federal Aviation Administration ….

18. Sargsyan, M. and A. Trchounian, *Development and validation of LC-MS/MS method for determination of branched chain amino acids and α-keto acids in human plasma.* International Journal of Mass Spectrometry, 2020. **453**: p. 116345.
